# Using Genetic Variation to Explore the Causal Effect of Maternal Pregnancy Adiposity on Future Offspring Adiposity: A Mendelian Randomisation Study

**DOI:** 10.1371/journal.pmed.1002221

**Published:** 2017-01-24

**Authors:** Rebecca C. Richmond, Nicholas J. Timpson, Janine F. Felix, Tom Palmer, Romy Gaillard, George McMahon, George Davey Smith, Vincent W. Jaddoe, Debbie A. Lawlor

**Affiliations:** 1 MRC Integrative Epidemiology Unit, University of Bristol, Bristol, United Kingdom; 2 School of Social and Community Medicine, University of Bristol, Bristol, United Kingdom; 3 The Generation R Study Group, Erasmus MC, University Medical Center Rotterdam, Rotterdam, the Netherlands; 4 Department of Epidemiology, Erasmus MC, University Medical Center Rotterdam, Rotterdam, the Netherlands; 5 Department of Pediatrics, Erasmus MC, University Medical Center Rotterdam, Rotterdam, the Netherlands; 6 Department of Mathematics and Statistics, Lancaster University, Lancaster, United Kingdom; University of Manchester, UNITED KINGDOM

## Abstract

**Background:**

It has been suggested that greater maternal adiposity during pregnancy affects lifelong risk of offspring fatness via intrauterine mechanisms. Our aim was to use Mendelian randomisation (MR) to investigate the causal effect of intrauterine exposure to greater maternal body mass index (BMI) on offspring BMI and fat mass from childhood to early adulthood.

**Methods and Findings:**

We used maternal genetic variants as instrumental variables (IVs) to test the causal effect of maternal BMI in pregnancy on offspring fatness (BMI and dual-energy X-ray absorptiometry [DXA] determined fat mass index [FMI]) in a MR approach. This was investigated, with repeat measurements, from ages 7 to 18 in the Avon Longitudinal Study of Parents and Children (ALSPAC; *n* = 2,521 to 3,720 for different ages). We then sought to replicate findings with results for BMI at age 6 in Generation R (*n* = 2,337 for replication sample; *n* = 6,057 for total pooled sample).

In confounder-adjusted multivariable regression in ALSPAC, a 1 standard deviation (SD, equivalent of 3.7 kg/m2) increase in maternal BMI was associated with a 0.25 SD (95% CI 0.21–0.29) increase in offspring BMI at age 7, with similar results at later ages and when FMI was used as the outcome.

A weighted genetic risk score was generated from 32 genetic variants robustly associated with BMI (minimum F-statistic = 45 in ALSPAC). The MR results using this genetic risk score as an IV in ALSPAC were close to the null at all ages (e.g., 0.04 SD (95% CI -0.21–0.30) at age 7 and 0.03 SD (95% CI -0.26–0.32) at age 18 per SD increase in maternal BMI), which was similar when a 97 variant generic risk score was used in ALSPAC.

When findings from age 7 in ALSPAC were meta-analysed with those from age 6 in Generation R, the pooled confounder-adjusted multivariable regression association was 0.22 SD (95% CI 0.19–0.25) per SD increase in maternal BMI and the pooled MR effect (pooling the 97 variant score results from ALSPAC with the 32 variant score results from Generation R) was 0.05 SD (95%CI -0.11–0.21) per SD increase in maternal BMI (*p*-value for difference between the two results = 0.05). A number of sensitivity analyses exploring violation of the MR results supported our main findings. However, power was limited for some of the sensitivity tests and further studies with relevant data on maternal, offspring, and paternal genotype are required to obtain more precise (and unbiased) causal estimates.

**Conclusions:**

Our findings provide little evidence to support a strong causal intrauterine effect of incrementally greater maternal BMI resulting in greater offspring adiposity.

## Abstract 2

Using a Mendelian randomization approach, Rebecca Richmond and colleagues find limited evidence for a causal effect of maternal pregnancy adiposity on future offspring adiposity during childhood and adolescence.

## Author Summary

**Why Was This Study Done?:**

- Being overweight or obese in pregnancy causes babies to be born larger.
- Whether these babies go into childhood and adolescence more overweight as a result of their mothers being overweight in pregnancy has not been fully established.
- We wanted to investigate whether genetically elevated body mass index (BMI) of mothers in pregnancy was causally associated with higher levels of fatness in their offspring in childhood and adolescence, over and above the association expected given genetic transmission of BMI-associated variants.

**What Did the Researchers Do and Find?:**

- We used information on 6,057 mother–child pairs from two prospective birth cohort studies, the Avon Longitudinal Study of Parents and Children (ALSPAC) and the Generation R study to assess this.
- Our findings provide little evidence to support the long-term impact of maternal BMI in pregnancy on the child’s risk of fatness in childhood and adolescence.
- Rather, most of the association between a mother’s BMI in pregnancy and her child’s fatness is explained by genetic transmission of BMI-associated variants.

**What Do These Findings Mean?:**

- These findings suggest that public health interventions directed at all family members and at different stages of the life course are likely to be important and are potentially more likely to halt the obesity epidemic than a focus on maternal overweight and obesity status in pregnancy.
- Although we found no large effects of being overweight or obese in pregnancy on offspring’s risk of fatness in later life, further studies with relevant data are required to determine whether there might be smaller effects that our study was unable to detect.

## Introduction

The developmental overnutrition hypothesis suggests mechanisms by which intrauterine conditions related to greater maternal adiposity might affect lifelong risk of offspring fatness [1]. Maternal body mass index (BMI) is positively associated with greater pregnancy-related increases in circulating glucose, lipids, and fatty acids [2,3], and in turn higher maternal gestational levels of these nutrients are associated with greater birth size [4–6]. In support of this hypothesis, strong evidence for a causal effect of greater maternal gestational adiposity and circulating fasting glucose, but not triglycerides, on birth weight and ponderal index at birth has recently been shown using a Mendelian randomisation (MR) approach [7]. As birth size is correlated with later size, it is possible that these effects will extend into later offspring postnatal life. In addition to this proposed tracking effect, it has been suggested that intrauterine exposure to higher levels of adiposity-related nutrients, such as glucose, results in permanent changes to offspring appetite control, neuroendocrine functioning, or energy metabolism, which subsequently result in greater adiposity in later life, irrespective of any effect on birth size [8–10]. Ascertaining whether greater maternal gestational adiposity results in greater offspring adiposity through intrauterine effects is important because if it does then that mechanism could result in acceleration of the obesity epidemic across generations [11,12] and would emphasise the importance of preconception or antenatal interventions in women of reproductive age to halt and reverse the obesity epidemic [13,14].

**Fig 1:**
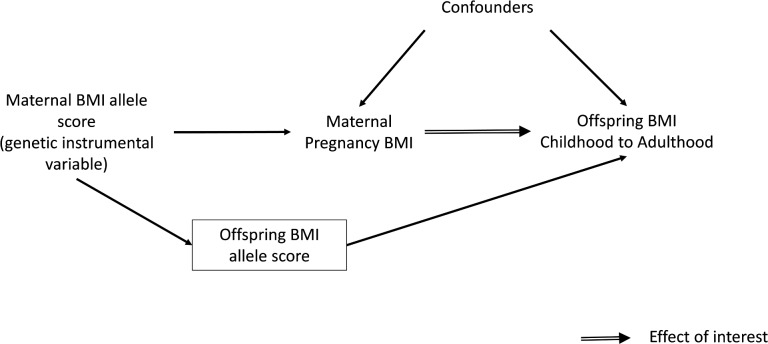
Intergenerational MR analysis to investigate a causal intrauterine effect of maternal BMI on offspring adiposity.

Several large cohort studies have shown that maternal pre- or early-pregnancy BMI is positively associated with offspring fatness, measured with BMI, waist circumference, or more direct assessments of fat mass, across the whole maternal BMI distribution [1,15–18]. However, due to the high heritability of adiposity and shared environmental and behavioural characteristics between mothers and their offspring, it is impossible to determine specific intrauterine effects from such studies [1,19,20]. An intergenerational MR design in which maternal genetic variants are used as instrumental variables (IVs) for environmentally modifiable intrauterine exposures, such as exposure to greater maternal adiposity, may be useful for providing insights into the causal effect of these exposures on later offspring outcomes (Fig 1) [21]. To our knowledge, this approach has only been used once to examine the causal effect of maternal BMI on offspring adiposity in childhood [22]. In that study, a variant in the *FTO* gene was used as an IV for pre-pregnancy BMI. The results suggested no causal effect of maternal gestational BMI on offspring dual-energy X-ray absorptiometry (DXA)-determined fat mass at age 10 y once offspring genotype was taken into account. However, the CI for the IV estimate was very wide, and it could not be statistically distinguished from the positive multivariable association of maternal pregnancy BMI with offspring fat mass. That MR study and, indeed, most of the conventional multivariable association studies have examined associations only in infancy or early childhood rather than into adulthood [1]. Examining associations at older ages is important because the potential for this mechanism to accelerate the obesity epidemic relates to maternal gestational adiposity, influencing their daughters’ adiposity during their reproductive years, such that they go into their pregnancies somewhat fatter and influence the next generation and so on through generations [1].

Our aim was to use MR to investigate the causal effect of intrauterine exposure to greater pre-/early-pregnancy maternal BMI on offspring BMI and fat mass using repeat measurements from childhood to early adulthood. The use of an allele score, generated from a large number of BMI genetic variants identified in genome-wide association studies (GWAS), can act as a stronger genetic instrument than a single variant [23,24]. Furthermore, we sought to replicate findings in an independent study to explore whether any results might be due to chance and, if results were replicated, to further increase study power by pooling results from the two studies.

## Methods

### Cohorts and Selection of Participants

We used data from mother–offspring pairs who participated in the Avon Longitudinal Study of Parents and Children (ALSPAC) [25,26] cohort in the main analysis and from the Generation R Study [27] in the replication analysis. In both studies, only singleton births were included because of the markedly different intrauterine growth patterns between singleton and multiple births. In ALSPAC, data were available on offspring BMI- and DXA-determined fat mass at multiple ages between 7 to 18 y (*n* = 2,521 to 3,720 mother–offspring pairs for different ages), whereas in Generation R (*n* = 2,337), offspring BMI at approximately 6 y of age was available.

### ALSPAC

The ALSPAC is a population-based prospective birth cohort study that enrolled 14,541 pregnant women residing in the former County of Avon, United Kingdom, with an expected delivery date between April 1, 1991 and December 31, 1992 [26,27]. The study website contains details of all available data through a fully searchable data dictionary (http://www.bristol.ac.uk/alspac/researchers/data-access/data-dictionary/)). Ethical approval for the study was obtained from the ALSPAC Ethics and Law Committee and the Local Research Ethics Committees. Of the 13,678 live-born singleton offspring in ALSPAC, genotype data were available for 5,206 mother-offspring pairs. For the main analysis, self-reported pre-pregnancy BMI was available for 4,629 of the included mother-offspring pairs, and of these, 3,720 offspring BMI measures were obtained at a clinic when the offspring were a mean age of 7.5 y (referred to as 7-y assessment). In ALSPAC, adiposity measures were assessed at five further follow-up clinics. These clinics took place when the offspring were mean ages 9.8, 11.7, 13.8, 15.4, and 17.8 y (henceforth referred to as 10, 12, 14, 16, and 18 y). The number of eligible individuals at these later clinics for inclusion in this study varied from 3,496 at age 10 to 2,521 at age 18 due to loss to follow-up at the later time points.

### Generation R

The Generation R study is a multiethnic population-based prospective cohort study from early pregnancy onward based in Rotterdam, the Netherlands [27]. All pregnant women living in the study area with a delivery date between April 2002 and January 2006 were eligible for enrolment. The study protocol was approved by the Medical Ethical Committee of the Erasmus Medical Centre, Rotterdam. The cohort includes 9,778 mothers and their children (9,749 live-born children). Of these, 8,880 (91%) women were enrolled during pregnancy and 8,633 had singleton live births. Genotype data were available for 3,909 mother–offspring pairs. For the main analysis, self-reported pre-pregnancy BMI was available for 3,199 of the included mother–offspring pairs and of these, 2,337 offspring BMI measures at a 6-y clinic (offspring mean age 6.2 y) were available.

### Anthropometry

Self-reported pre-pregnancy weight and height were obtained for the mothers in both cohorts during pregnancy and used as the main exposure. In both studies, recorded weight at the first antenatal clinic visit correlated very highly with their maternal report of pre-pregnancy weight at recruitment (correlation coefficients = 0.96). For each study, age (in 1-y categories) z-scores for maternal BMI were derived using internal standardisation. In ALSPAC and Generation R, offspring height and weight were measured at research clinics and used to calculate offspring BMI. For each study, internally standardised sex and age (in month categories) z-scores for offspring BMI and (in ALSPAC only) DXA-determined fat mass index (FMI) were calculated.

### ALSPAC

After enrolment, the mother was asked to report her height and pre-pregnancy weight in a questionnaire administered at 12 wk gestation, from which pre-pregnancy BMI was calculated (as weight [in kilograms] divided by height [in meters)]squared). The correlation of pre-pregnancy weight obtained by questionnaire and weight measured at the first antenatal visit (10–12 wk gestation) was 0.96. The women’s partners (fathers of the children) reported their own heights and weights in questionnaires completed at the same time as the mothers; these were used to determine their BMI.

From age 7 onwards, offspring were invited to follow-up clinics where anthropometry was measured. Weight and height were measured with the child in light clothing and without shoes. Weight was measured to the nearest 0.1kg using a Tanita Body Fat Analyser (Model TBF 305; Tanita UK Limited, Viewsley, UK) and height to the nearest 0.1cm using a Harpenden Stadiometer (Holtain Limited, Dyfed, UK). BMI (kg/m^2^) was then calculated as weight (in kilograms) divided by height (in meters) squared. The age of the child when they attended the research clinic was recorded in months.

From the 9-y clinic onwards, total fat mass (kg) and total lean mass (kg) were assessed by whole body DXA with the use of a Lunar Prodigy DXA scanner (GE Medical Systems Lunar, Madison, WI). The scans were visually inspected and realigned where necessary. Once complete, the tester examined the scan to ensure its quality and, if necessary, repeated the scan. FMI was subsequently calculated as total fat mass (in kilograms) divided by height (in meters) squared.

### Generation R

Information about maternal weight just before pregnancy was obtained by questionnaire, and height was measured without shoes and heavy clothing on enrolment, from which BMI (kg/m^2^) was calculated. The correlation of pre-pregnancy weight obtained by questionnaire and weight measured at the first antenatal visit was 0.96. Partner’s height (cm) and weight (kg) were also measured at enrolment.

When the children were age 5 onwards, they were invited to participate in a hands-on assessment of anthropometry. Their height and weight without shoes and heavy clothing were measured and used to calculate BMI. Weight (kg) was measured using a mechanical personal scale (SECA, Almere, The Netherlands) and height (m) was determined in standing position using a Harpenden stadiometer (Holtain Limited, Dyfed, UK). The age of the child when they attended the research clinic was recorded in months.

### Genotyping

The genotyping procedures and details of the BMI SNPs obtained from genotyping for the mothers and offspring in the two cohorts are outlined below.

### ALSPAC

ALSPAC mothers were genotyped using the Illumina 660K quad SNP chip (Illumina Inc., San Diego, CA, US) at the Centre National de Genotypage, Paris. PLINK software [28] (v1.07) was used to carry out quality control (QC) measures. Individuals with incorrect sex assignments, unusual genome-wide or X-chromosome heterozygosity, disproportionate levels of individual missingness (>5%), evidence of cryptic relatedness (>12.5% identity-by-descent [IBD]), or those of non-European ancestry determined from multidimensional scaling analysis seeded with individuals from the International HapMap project [29] were excluded. The resulting dataset consisted of 8340 individuals [30].

SNPs with a minor allele frequency (MAF) of <1%, a call rate of <95%, or those not in Hardy–Weinberg equilibrium (HWE, *p* < 1 x 10^−6^) were removed [30]. Imputation of the directly genotyped data has been conducted with MaCH (v1.0.16) Markov Chain Haplotyping software [31,32] using CEU individuals from HapMap phase 2 (release 22) as a reference set for autosomal imputation [29].

ALSPAC offspring were genotyped using the Illumina HumanHap550 quad genome-wide SNP genotyping platform (Illumina Inc., San Diego, CA, US) by the Wellcome Trust Sanger Institute (Cambridge, UK) and the Laboratory Corporation of America (Burlington, NC, US). A similar QC procedure to that performed in the ALSPAC mothers was carried out. Individuals with incorrect sex assignments, extreme heterozygosity (<0.320 and >0.345 for Wellcome Trust Sanger Institute data and <0.310 and >0.330 for LabCorp data), disproportionate levels of individual missingness (>3%), evidence of cryptic relatedness (>10% IBD), or those of non-European ancestry were excluded. The resulting dataset consisted of 8,365 individuals. [33,34]

SNPs with a MAF of <1%, a call rate of <95%, or those not in HWE (*p* < 5 x 10^−7^) were removed [33,34]. Imputation of the directly genotyped data has been conducted in the same way as the mothers.

Established BMI variants [35,36] in the ALSPAC mothers and offspring were extracted from the data set imputed using the HapMap individuals as a reference set (in which all genotypes for BMI were present).

### Generation R

Genome-wide data are currently not available for the mothers in Generation R, but DNA is available for genotyping in candidate gene or replication studies. Therefore, custom genotyping of 32 SNPs identified in a large-scale GWAS meta-analysis for BMI [35] was carried out by LGC Genomics (formerly Kbiosciences) using a Taqman allelic discrimination assay (Applied Biosystems, Foster City, CA) and Abgene QPCR ROX mix (Abgene, Hamburg, Germany) with a call rate of 99.3%, duplicate concordance of 99.8%, and all considered SNPs found to be in HWE, with the exception of rs4836133 (*ZNF608*; *p* = 3 x 10^−15^). To confirm the accuracy of the genotyping results, 276 randomly selected samples were genotyped for a second time using the same method, with an error rate of <1%.

Genome-wide genotyping in the offspring was performed using either the Illumina HumanHap 610 or 660 Quad chips (Illumina Inc., San Diego, US) depending on time of DNA collection [37,38]. QC of the genotype and imputation process was performed in the study as previously described [38,39]. In brief, individuals with duplicate detection, low call rates (<97.5%), sex mismatches, and high heterozygosity (>4 standard deviations [SDs]) were excluded, and SNPs with low call rates (<98.0%), not in HWE (*p* < 1 x 10^−6^), with a low MAF (< 0.1%) or those with differential missingness between the two chips (*p* < 1 x 10^−7^) were excluded.

Ethnic composition of the sample was estimated by Identity-By-State analysis using principal components analysis (PCA) seeded with International HapMap Phase 2 release 22 individuals [29]. Participants were defined as being of non-European ancestry when they deviated more than 4 SDs from the CEU panel mean value in any of the first four principal components. Cryptic familial relationships were identified through IBD analysis.

MaCH imputation [32] of the offspring genetic data was done using both HapMap Phase 2 release 22 [29] and 1000 Genomes Phase 3 [40] reference panels, using all available haplotypes from the different populations in a “cosmopolitan” approach [38]. Established BMI variants [35] were extracted from the 1000 Genomes imputed data set due to missingness of three of the SNPs in the HapMap phase 2 data set.

From the maternal and offspring genotype data, weighted BMI allele scores were generated using 32 independent variants that have been shown to be reliably associated with BMI in both cohorts [41]. In ALSPAC, it was also possible to generate an additional allele score composed of 97 independent variants associated with BMI in a more recent GWAS [36], as ALSPAC has full genome-wide data on both mothers and offspring. Allele scores were derived using the dose of the effect (BMI-increasing) allele at each SNP, which was first weighted by the effect size of the variant in GWAS [36,41] and then summed:

$$Weighted\;BMI\;score=w_{1}\times {SNP}_{1}+w_{2}\times {SNP}_{2}+\cdots \;w_{n}\times {SNP}_{n}$$

where w is the weight (i.e., the beta-coefficient of association of the SNP with BMI from the published GWAS) and SNP is the dosage of BMI-raising alleles at that locus (i.e., 0, 1, or 2 BMI-raising alleles). The weights used are provided in S1 Table. The score was then rescaled to reflect the average number of BMI-increasing alleles carried by an individual using the formula described in Lin et al. [42]:

$$Rescaled\;weighted\;BMI\;score=\frac{Weighted\;score\;\times \;Number\;of\;SNPs\;available}{Sum\;of\;weights\;of\;available\;SNPs}$$

### Other Variables

Parental social class and education, maternal smoking during pregnancy, parity, and paternal BMI were considered as potential confounding factors in the multivariable regression analyses. Ethnicity was also considered as a potential confounder in Generation R, a multiethnic cohort (in ALSPAC, 95% of participants were white European origin). Details of how each of these confounders were assessed in the two cohorts are outlined below.

### ALSPAC

Parity, defined as the number of previous pregnancies resulting in a live or stillbirth, was recorded in a questionnaire completed at 18 wk gestation. In a questionnaire completed at 32 wk of gestation, mothers recorded the occupation and education of themselves and their partners. Highest occupation of the mother or their partner was used to allocate family social class groups (classes I [professional occupations], II [managerial and technical occupations], III non-manual [skilled non-manual occupations], III manual [skilled manual occupations], IV [partly skilled occupations], V [unskilled occupations]) using the 1991 British Office of Population Censuses and Surveys classification. Highest educational qualifications of the mother and father were treated as separate variables, and each was collapsed into one of four categories: education up to age 16 y with vocational training or certificate of secondary education, education up to age 16 y with general certification of education (ordinary level), education up to age 18 with general certificate of education (advanced level), and university degree. Information on mothers’ smoking status during pregnancy was obtained in questionnaires administered at 18 and 32 wk of gestation. Data were used to generate a categorical variable: never smoked during pregnancy, smoked in early pregnancy only, and smoked throughout pregnancy. Maternal age at delivery was derived from the mother’s date of birth, which was recorded at the time of recruitment, and the date of birth of her offspring. Offspring sex was recorded in the delivery room and abstracted from obstetric records and/or birth notifications.

### Generation R

Information about the mother’s parity, defined as the number of times that the woman had given birth to a foetus with a gestational age of 24 wk or more, was obtained by questionnaire at enrolment. Information about household income (euro/month) was obtained from a questionnaire administered during pregnancy. The highest completed education level (primary school, secondary school, higher education) for both mothers and fathers was obtained from questionnaires administered at enrolment. Information about maternal smoking in pregnancy was assessed by questionnaire in each trimester and was categorised into never smoked during pregnancy, smoked in early pregnancy only, and smoked throughout pregnancy. Maternal age was obtained in the questionnaire administered at enrolment. Using this and information about the gestational age of the foetus at enrolment and gestational age at birth, mother’s age at delivery was derived. Offspring sex was obtained from midwife and hospital registries at birth.

### Statistical Analysis

We examined multivariable regression associations and MR effects of maternal pre-pregnancy BMI on offspring BMI at ages 6–7 y in the two cohorts and both BMI and DXA fat mass at all ages with available data up to the age of 18 y in ALSPAC. We were unable to find any other study with relevant data on maternal and offspring genetic variants, maternal pre- or early-pregnancy BMI, and offspring BMI or fat mass in adolescence/early adulthood and so could not explore replication of ALSPAC findings at older ages. Results are presented in males and females combined as point estimates and looked very similar in both sexes, and there was no strong statistical evidence for an interaction by sex in either the multivariable or MR analyses (all *p*-values >0.07).

We followed an analysis plan, which was written before analysing any data for the main analyses, while some sensitivity analyses were carried out post hoc (S1 Appendix). All analyses were undertaken using Stata (Stata Corp, TX, US), version 13.

### Analysis of Effect of Maternal Pre-pregnancy BMI on Offspring Adiposity through Childhood to Young Adulthood in ALSPAC

Multivariable linear regression was performed to examine the association of maternal pre-pregnancy BMI with offspring BMI from ages 7 to 18 and FMI from ages 10 to 18. In the first model, maternal age, offspring age, and sex were controlled in the standardised exposure and outcome. In the second model, we additionally adjusted for potential confounding by parental social class, parental education, maternal smoking during pregnancy, parity, and paternal BMI.

MR analysis was first carried out using the maternal weighted BMI allele score composed of 32 SNPs as an IV for her pre-/early-pregnancy BMI to assess its causal effect on offspring BMI (from ages 7 to 18) and offspring FMI (from ages 10 to 18). Both multivariable and IV approaches examined the same relationship, i.e., the SD change in outcome per 1 SD increase in maternal pre-/early-pregnancy BMI.

We used two-stage least squares (TSLS) IV analysis for the MR approach. We assumed an additive genetic model as supported by the original GWAS [41]. The strength of the IV was assessed by examining the R^2^ and F-statistics from the first stage regression for each analysis [43]. Maternal age, offspring age, and sex were controlled in all MR analyses by the use of exposure and outcome measurements standardised for these characteristics. Despite evidence that the lifestyle and socioeconomic characteristics that commonly confound observational studies are randomly distributed with respect to genotype [44], we tested this assumption in our study by examining associations between the allele score and potential confounders of the observational association [45].

To obtain a causal estimate of the intrauterine influence of maternal BMI on offspring BMI, it is important to adjust for offspring genotype to exclude the possibility of another independent pathway between the genetic instrument and outcome, i.e., through genetic transmission from the mother to their offspring (Fig 1). This was achieved by adding the offspring weighted BMI allele score to the IV models. A z-test was used to test for a difference between this MR IV analysis and the confounder-adjusted multivariable regression analysis, with evidence for a difference between the two being indicative of the possibility of unobserved confounding in the multivariable analysis. The z-statistic was calculated by estimating the covariance between the multivariable regression and IV estimates using a bootstrapping procedure.

We generated an additional weighted allele score from 97 genetic variants, using SNP-specific weights taken from the recent GWAS meta-analysis in which they were all identified [36], in order to explore whether our main findings were consistent when using this score, with potentially greater power.

### Replication in an Independent Cohort and Meta-Analysis

We undertook multivariable regression and MR IV analyses in the replication Generation R Study using the same methods as described above for ALSPAC, with the additional adjustment for ethnicity, by including the first 20 principal components obtained from the offspring genotype data as covariates in both analyses. We were only able to complete IV analyses using the 32-SNP weighted allele score in this cohort.

We used a fixed effects meta-analysis to combine the multivariable regression results from ALSPAC and Generation R and used Cochran’s Q test and the I^2^ statistic to explore heterogeneity between the results from these two cohorts [46]. We took a similar approach to pooling the MR IV results. We did this by pooling the results from the two stages of the IV analyses separately: (i) the maternal allele score (with and without adjustment for offspring allele score) association with maternal BMI (first regression) and (ii) the maternal allele score (with and without adjustment for offspring allele score) association with offspring BMI at age 6 (second regression). We then combined these two pooled estimates to obtain the IV MR causal effect using the ratio estimate, i.e., the pooled results of (ii) ÷ the pooled results of (i) [47]. The standard errors of these estimates were calculated using a Taylor series approximation [48]. We pooled results using the 32-SNP scores generated in both studies and also results using the 97-SNP score in ALSPAC with the 32-SNP score in Generation R. We compared the pooled offspring allele score-adjusted MR IV results with the pooled confounder-adjusted multivariable estimates using a z-test and bootstrapping, as described above for ALSPAC.

To provide results that are more interpretable for clinical and public health use, we converted the results on the SD scale to BMI units by multiplying them by a representative value of the SD of pre-pregnancy BMI (3.7 kg/m^2^) and offspring BMI (2.0 kg/m^2^) taken from the Discovery and largest study (ALSPAC).

### Sensitivity Analyses

In the MR analysis, it is necessary to adjust for offspring BMI allele score to separate the influence of genetic inheritance from the intrauterine effect of maternal adiposity during pregnancy. However, as was highlighted in a response to the previous paper where this method was used [49], adjustment may introduce a form of bias known as collider bias in estimating the exposure–outcome association. This is because by adjusting for offspring allele score in the MR IV analyses, we may induce an association via paternal genetic variants (which we do not have data on; S1 Fig). We explain this possibility in more detail in the appendix (S1 Appendix) and also describe how we undertook simulation studies to explore the likelihood of this biasing our main results with adjustment for offspring allele score. Furthermore, an alternative method for avoiding possible collider bias is to use only the maternal non-transmitted alleles [50]. We explored the use of this approach within ALSPAC and present results in the appendix (S1 Appendix) only given the relatively low statistical power associated with this method.

A high degree of heterogeneity between causal estimates of the individual SNPs comprising the allele scores could indicate violation of the MR assumption that there is no pleiotropy. We therefore performed inverse-variance weighted (IVW) meta-analysis of the individual SNP estimates in both ALSPAC and Generation R and calculated Cochran’s Q and I^2^ statistic to estimate the degree of heterogeneity in the fixed effects meta-analysis [51]. We also investigated potential bias due to pleiotropy by performing MR Egger regression [52] for the model adjusted for offspring allele score. The intercept in this analysis provides a test for overall directional pleiotropy, and the coefficient provides a valid causal estimate in the presence of pleiotropy. Results were obtained for both ALSPAC (using both sets of 32 SNPs and 97 SNPs) and Generation R (using the set of 32 SNPs). The intercepts and slopes were meta-analysed and compared with those obtained using IVW of the individual SNPs. Analysis was performed using the mrrobust Stata package [53].

Lastly, we investigated possible nonlinearity of the association in both the multivariable regression and MR analyses by overlaying a nonparametric loess smoother and a line of best fit on an augmented partial residual plot.

## Results

Table 1 shows the key characteristics of the Discovery and Replication cohort.

**Table 1:** Characteristics of the offspring and their mothers in the Discovery and Replication cohorts.

	ALSPAC (Discovery)	Generation R (Replication)	Generation R (Europeans)*
*n*	3,720	2,337	1,280
Males (%)	48.6%	49.6%	49.4%
Offspring age in months (SD)	89.6 (1.9)	74.3 (6.1)	73.2 (4.6)
Offspring birth weight in gs (SD)	3,465 (511)	3,479 (507)	3,561 (506)
Offspring weight in kgs (SD)	25.6 (4.4)	23.2 (4.1)	22.8 (3.4)
Offspring height in cm (SD)	125.7 (5.4)	119.5 (5.9)	119.5 (5.4)
Offspring BMI in kg/m^2^ (SD)	16.2 (2.0)	16.2 (1.8)	15.9 (1.4)
Maternal BMI in kg/m^2^ (SD)	22.9 (3.7)	23.5 (4.1)	23.2 (3.8)

*Generation R is a multiethnic cohort; these are characteristics for those of European origin only (as defined by 4 SDs from the HapMap CEU panel mean value for all four principal components from the genetic data).

### Analyses from Childhood to Early Adulthood in ALSPAC

The sample size at each age from 7 to 18 decreased as a result of loss to follow-up, but the proportion of males and females and the distribution of birth weight (of those remaining in the cohort) were similar in each age group (S2 Table). Height, weight, BMI, fat mass, FMI, and lean mass increased with increasing age from 7 to 18 y as expected (S2 Table).

Maternal BMI was associated with characteristics that a priori we considered to be likely confounders (S3 Table). With one exception, the maternal BMI allele score was not associated with these confounders. There was a weak inverse association with paternal education (suggesting that on average each category increase of paternal education was associated with a -0.03 [95% CI -0.07–0.00] SD change in the weighted allele score [S3 Table]). The maternal BMI allele score was normally distributed and was robustly associated with maternal pre-pregnancy BMI, explaining 2.2% of the variation in BMI (S4 Table**),** and with first-stage F-statistics for each of the MR analyses all being >45 (S5 Table).

Table 2 shows the confounder-adjusted multivariable associations of maternal pre-pregnancy BMI with offspring BMI and FMI at each age and the equivalent MR results (with adjustment for offspring allele scores). S6 and S7 Tables show more detailed results, including the multivariable results unadjusted for offspring allele scores and MR results. In confounder-adjusted multivariable regression, a 1 SD (equivalent to 3.7 kg/m^2^) higher age-adjusted maternal BMI was associated with a 0.25 (0.21, 0.29) SD higher offspring BMI at age 7 and a 0.33 SD higher offspring BMI (0.28, 0.37) at age 18 (equivalent of 0.56 and 0.76 kg/m^2^, respectively; Table 2). Equivalent results for the genetic IV analyses with adjustment for offspring allele score were 0.04 (-0.21, 0.30) SD at age 7 and -0.03 (-0.32, 0.26) SD at age 18 (Table 2). Results for FMI from ages 10 to 18 were similar for both the multivariable regression and MR results to those seen with BMI (Table 2). We further evaluated, in a post hoc manner, the impact of additionally adjusting for maternal and offspring allele score in the multivariable regression models and found that these additional adjustments did not substantially change the point estimates for mean difference (S14 Table).

**Table 2:** Confounder-adjusted multivariable and genetic IV (MR) associations of maternal pregnancy BMI with offspring BMI and FMI from ages 7 to 18 in ALSPAC (Discovery sample).

Offspring outcome	Confounder^a^-adjusted multivariable regression results		MR (genetic IV maternal allele score-adjusted for offspring allele score) results		*p*-difference^b^
	*n*	Difference in mean offspring outcome (SD) per 1SD increase maternal BMI (95%CI)	*n*	Difference in mean offspring outcome (SD) per 1SD increase maternal BMI (95%CI)	
BMI age 7	2,565	0.25 (0.21–0.29)	3,720	0.04 (-0.21–0.30)	0.13
BMI age 10	2,507	0.31 (0.27–0.35)	3,657	0.03 (-0.23–0.29)	0.03
BMI age 12	2,411	0.32 (0.29–0.36)	3,496	0.00 (-0.26–0.26)	0.02
BMI age 14	2,254	0.32 (0.28–0.36)	3,227	-0.07 (-0.34–0.20)	0.01
BMI age 16	1,979	0.34 (0.30–0.39)	2,806	-0.10 (-0.41–0.20)	0.003
BMI age 18	1,798	0.33 (0.28–0.37)	2,521	-0.03 (-0.32–0.26)	0.01
FMI age 10	2,413	0.30 (0.26–0.33)	3,495	0.13 (-0.13–0.39)	0.221
FMI age 12	2,375	0.31 (0.27–0.35)	3,444	0.04 (-0.22–0.30)	0.053
FMI age 14	2,233	0.30 (0.26–0.34)	3,192	0.03 (-0.23–0.29)	0.043
FMI age 16	1,927	0.33 (0.29–0.38)	2,715	-0.10 (-0.40–0.21)	0.001
FMI age 18	1,739	0.32 (0.27–0.37)	2,430	0.03 (-0.27–0.32)	0.033

In all analyses, offspring BMI has been standardised on their sex and age and maternal BMI has been standardised on her age.

^a^The following were adjusted for in multivariable regression analyses: parental social class, parental education, maternal smoking during pregnancy, parity, and paternal BMI.

^b^Testing the null hypothesis that the multivariable regression analysis results do not differ from the MR results.

Abbreviations: BMI, body mass index; FMI, fat mass index.

There was strong statistical evidence that the MR IV analysis results differed from the multivariable regression analysis results, with the exception of offspring BMI at age 7 and FMI at age 10, where the IV associations had point estimates that were smaller than the multivariable regression analysis results but were statistically consistent with those results (Table 2). Results were similar when the 97-SNP BMI allele score was used as an IV (S8 and S9 Tables).

### Replication and Meta-Analysis with an Additional Independent Cohort

Maternal BMI was associated with potential confounders in Generation R (S10 Table). The maternal BMI allele score was normally distributed with similar mean and SD as in ALSPAC and was robustly associated with maternal pre-pregnancy BMI (S4 Table). Similar to ALSPAC, maternal BMI allele score was weakly inversely related to paternal education in Generation R but not to other observed confounders (S10 Table).

Age- and sex-adjusted results were similar to those in ALSPAC, although there was evidence of heterogeneity in the confounder-adjusted multivariable regression results (I^2^ = 82%; S2 Fig). Pooling results from both studies showed that a 1 SD (equivalent of 3.7 kg/m^2^) increase in age-adjusted maternal BMI was associated with a 0.22 (0.19, 0.25) SD (equivalent of 0.44 [0.38, 0.50] kg/m^2^) increase in offspring BMI in the confounder-adjusted model. The maternal BMI allele score was similarly positively associated with offspring BMI in both studies but with adjustment for offspring allele score the associations attenuated to the null, with no evidence for heterogeneity between the estimates (S3 Fig).

**Fig 2:**
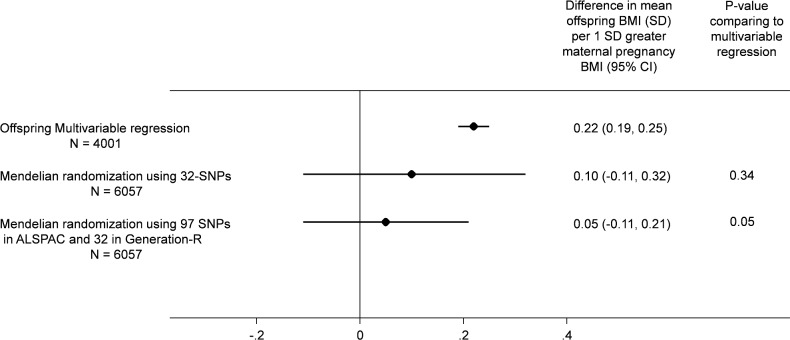
Relationship of maternal pregnancy BMI with offspring BMI at age 7: pooled ALSPAC and Generation-R multivariable and MR analyses.

There were similar associations of the maternal allele score with maternal BMI and with offspring BMI between the two cohorts (ALSPAC and Generation R; S3 Fig). Although the MR effect when pooling the offspring-adjusted results based on the 32-SNP allele score in both cohorts appeared weaker than our confounder-adjusted multivariable estimate, 0.10 (-0.11, 0.31) SD (equivalent of 0.20 [-0.22, 0.62] kg/m^2^) versus 0.22 (0.19, 0.25) SD (equivalent of 0.44 [0.38, 0.50) kg/m^2^] per 1 SD greater maternal pre-pregnancy BMI, there was no strong statistical evidence that these two estimates differed from each other (*p*(diff) = 0.34; Fig 2). When we pooled the MR results using the 97-SNP allele score from ALSPAC with those from Generation R using the 32-SNP allele score, the point estimates were closer to the null and were more precisely estimated (0.05 [-0.11, 0.21] SD per 1 SD greater maternal pre-pregnancy BMI), and there was increasing statistical evidence for a difference between these MR results and the confounder-adjusted multivariable estimate (*p*(diff) = 0.05; Fig 2).

All results are difference in mean offspring BMI at age 7 (SD units) per increase in 1 SD maternal pregnancy BMI pooling the results from ALSPAC (Discovery cohort) with those from Generation R Study (Replication cohort). In all analyses, offspring age and sex are controlled for through standardisation of the offspring BMI, and maternal age is controlled for by standardisation of BMI on age. The multivariable regression analyses results are controlled for parental social class, parental education, maternal smoking during pregnancy, parity, and paternal BMI and in Generation R only for ethnicity (adjusting for top 20 principal components of offspring genome-wide array data). The MR results are adjusted for offspring allele score and in Generation R only for ethnicity (adjusting for top 20 principal components of offspring genome-wide data).

Given the weak association of the maternal allele score with paternal education in both cohorts, we re-ran all of the IV analyses with additional adjustment for paternal education. Additional adjustment for paternal education did not alter the null MR estimate following adjustment for offspring allele score (S4 Fig). In addition, analyses restricted to those of European ancestry in Generation R were similar to those including all Generation R participants (S5 Fig).

### Sensitivity Analyses

Our investigation of the possibility of introducing bias via paternal genotype suggested that the offspring allele score-adjusted MR estimate was the least bias result of the causal effect of maternal pre-pregnancy BMI on offspring BMI. Although the inability to also adjust for paternal genetic variants meant this was somewhat biased towards the null, it was unlikely to have markedly changed our findings (S1 Appendix and S11 Table). Furthermore, we explored the use of the non-transmitted (to offspring) haplotype approach [50] in ALSPAC and both the 32-SNP and 97-SNP haplotype scores revealed that the non-transmitted maternal haplotype score did not show strong evidence of association with offspring while the transmitted maternal haplotype score was strongly associated, indicating the expected offspring genetic influence on their own BMI but providing little support for a maternal BMI intrauterine effect on offspring BMI (S1 Appendix and S12 Table).

Results of the IVW MR approach showed estimates approximately equal to the TSLS allele score approach. Furthermore, this approach showed no clear evidence for heterogeneity of effect estimates of the individual SNPs comprising the score in ALSPAC or Generation R (I^2^ = 0%; S6 Fig, S13 Table). In addition, the MR Egger method gave no indication of directional pleiotropy influencing the results of the MR analysis (intercept = 0.005 [-0.003, 0.013], *p* = 0.13) for the meta-analysis of 97 SNPs in ALSPAC and 32 SNPs in Generation R) and provided evidence for a lack of consistent causal effect of maternal BMI on offspring BMI in the models adjusted for offspring genotype (coefficient = 0.08 SD [-0.12, 0.28], *p* = 0.07) in the meta-analysis of estimates from ALSPAC and Generation R, although there was some degree of heterogeneity between the two studies (I^2^ for slope = 70%; S6 Fig, S13 Table). There was no strong evidence for departure from linearity in the relation of maternal BMI with offspring BMI in either multivariable or MR analyses (S7 Fig and S8 Fig).

## Discussion

**Fig 3:**
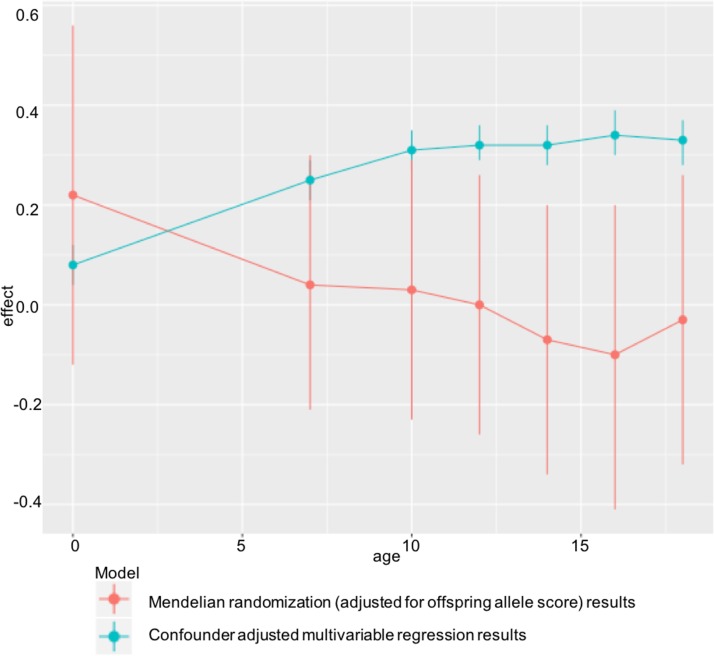
Diminishing causal effect of developmental overnutrition across the life course. Multivariable and IV effect estimates from ALSPAC at ages 7–18 (Table 2) compared with those obtained when investigating the effect of maternal BMI on offspring ponderal index (kg/m^3^) at birth in the same cohort.

In ALSPAC, we found positive associations of maternal pre-pregnancy BMI with offspring BMI and FMI at all ages from childhood to early adulthood in confounder-adjusted multivariable regression analyses. However, we found no evidence for these associations being causal in MR IV analyses using a weighted allele score of 32 (or 97) genetic variants known to be robustly associated with BMI. There was statistical evidence that the confounder-adjusted multivariable analyses differed from the offspring genetic variant-adjusted MR analyses, and results were virtually identical when we used FMI measured by DXA scan. ALSPAC findings were replicated in the independent Generation R Study with BMI assessed at age 6 y. Taken together, these results do not support an important causal intrauterine effect of greater maternal BMI on later offspring adiposity. This is in contrast to evidence of a causal effect of greater maternal adiposity on birth weight and ponderal index at birth identified using MR in a previous study that included both of the cohorts used here [7], potentially indicating a diminishing effect of this intrauterine exposure over the life course (Fig 3).

Findings of a null causal effect from MR analysis in both ALSPAC (at all ages from childhood to early adulthood) and Generation R (in childhood) are consistent with most of the negative control studies, which show a similar magnitude of association between maternal pre-pregnancy BMI and offspring BMI as that between paternal BMI and offspring BMI [54–59], although none of those studies assessed outcomes in early adulthood. They are also consistent with a large within-sibling analysis, which suggested that shared familial characteristics confounded the positive maternal early-pregnancy BMI–offspring BMI association at age 18, as this disappeared when analyses were conducted within siblings with close matching of shared family characteristics [60]. However, that study was performed in male offspring only. Lastly, the results are consistent with the previous MR study in ALSPAC conducted using the *FTO* variant only as an IV for maternal BMI and examining offspring BMI- and DXA-determined fat mass at age 10 [22]. By combining a large number of genetic variants in an allele score, in this study, we have increased statistical power and shown a difference between the multivariable and IV analysis in ALSPAC at multiple ages (from childhood to early adulthood), which was not the case with the previous ALSPAC publication. We have also shown independent replication of our findings in Generation R, investigated the use of multiple instruments, and tested for potential pleiotropic effects.

For the MR analysis, we thoroughly investigated any violation of the main assumptions of this approach, which are that the IV is robustly associated with the exposure of interest, not related to confounding factors of the exposure–outcome association, and not related to the outcome independent of its effect on the exposure [45].

The allele scores derived from established variants for BMI were strongly associated with the exposure of interest (maternal BMI) in the full cohorts, and large F-statistics indicated sufficient strength as IVs, enabling a more precise assessment of the effect of maternal BMI than was previously possible with the use of a single genetic variant [22].

The allele scores were not consistently associated with a range of socioeconomic factors, and this illustrates a key strength of the MR approach. In addition, by adjusting for offspring genotype, intergenerational MR is able to distinguish between causal maternal effects on offspring adiposity from genetic transmission of adiposity variants.

The existence of pleiotropy, in which a genetic instrument has an effect on an outcome (offspring BMI) independent of its effect on the exposure (maternal BMI), would have implications for the assumptions made in the MR analysis. Similarly, if a genetic variant in the score was in linkage disequilibrium with another genetic variant that influences the outcome through a pathway that is unrelated to the exposure, this could bias the causal estimate. The fact that some BMI variants relate to glucose and other metabolites [61,62] is an example of type 2 or spurious pleiotropy and is unlikely to bias the IV effects [63]. We have attempted to control for the effect of offspring genotype, which is related to maternal genotype, on offspring BMI by adjusting for offspring genotype. It is hard to determine a real pleiotropic effect that would produce the null effects seen following this adjustment. In addition, the consistency of IV estimates obtained using two allele scores in this study suggests that pleiotropy is unlikely. Furthermore, there was no strong evidence for heterogeneity of individual SNP estimates comprising the allele scores, and the MR Egger method gave no indication of directional pleiotropy in ALSPAC or Generation R.

Although maternal genetic variants are being used as an unconfounded proxy for an intrauterine exposure, these variants will also impact on maternal BMI after pregnancy and so any causal effect, had it been identified, might also incorporate postnatal effects. For example, mothers who are genetically predisposed to have a higher BMI might influence their offspring’s adiposity through postnatal characteristics such as maternal feeding behaviours [64]. The heterogeneity observed in the confounder-adjusted multivariable models could represent variation in prevailing postnatal environmental contexts, for example cultural influences on parents’ child-feeding behaviour [65]. However, the MR effects were null in both ALSPAC and Generation R, suggesting that the causal effect of postnatal maternal BMI on offspring adiposity is at most very small. The weak inverse association of maternal BMI allele scores with their partners (but not their own) education that we saw in both cohorts is intriguing and worth further exploration, but adjusting for this did not alter our MR results.

It is important to adjust for offspring genotype in the MR analyses, but in doing so we might introduce a path between maternal genotype and offspring BMI via paternal genotype. Our simulations suggested that this would result in a weak bias towards the null, but this bias would not fully explain the null effects we observe as the results in the simulation study with adjustment for offspring allele score were close to the true simulated result (S11 Table). Furthermore, our findings were corroborated with results from the non-transmitted haplotype analysis [50], applied in ALSPAC, which revealed that the non-transmitted maternal haplotype score did not show strong evidence of association with offspring BMI.

In the MR meta-analysis using the 97-SNP allele score in ALSPAC and the 32-SNP allele score in Generation R, with adjustment for offspring genotype (*n* = 6,057), we had 82% power to detect a causal effect the size of that seen in the multivariable analysis controlled for age and sex (0.28 SD) with a two-sided α = 0.05 in our MR analysis. Therefore, we were adequately powered to detect an effect the size of that seen observationally, and the heterogeneity we see between the multivariable and MR results is unlikely to be due to chance. However, we would need a much larger number of participants to completely rule out evidence for a weaker causal effect.

Furthermore, power was limited for some of the sensitivity tests such as the evaluation of SNP heterogeneity, the MR Egger analysis, and the evaluation of heterogeneity in causal estimates between ALSPAC and Generation R. Further studies with relevant data on maternal, offspring, and paternal genotype are required to obtain more precise (and unbiased) causal estimates.

Further limitations to the MR analysis include the possibility of population stratification and canalisation [66]. Although in ALSPAC population stratification is unlikely because the participants are unrelated individuals of European ancestry, the Generation R Study is a multiethnic cohort. However, attempts made to adjust for population stratification by including principal components and analyses restricted to those of European ancestry were similar on the whole (S3 Fig). Developmental canalisation, in which systems develop differently to counterbalance the effects of a particular genotype, may pose a problem for conventional MR. However, this is less of an issue in intergenerational MR analysis. This is because when maternal genotype is used as an indicator of the intrauterine environment, then this will only influence the developmental environment of the offspring through the exposure of interest and not by competing mechanisms [67].

Maternal pre-pregnancy BMI was self-reported in both cohorts. Although these measures of self-report have been shown to correlate strongly with measured BMI in early pregnancy [68–70], the possibility of systematic under-reporting, for example, if those who are heavier systematically under-report their weight, might bias findings. However, previous analysis in ALSPAC has shown that misreporting is similar for the majority of participants and is not markedly influenced by mean weight [71]. Furthermore, the magnitude of the association of the weighted allele score with maternal BMI in this study was similar to that seen for its relationship to BMI based on measured weight and height from other studies [7].

A general limitation of the longitudinal measures in ALSPAC is loss to follow-up of the sample over time, from 3,720 participants at age 7 to 2,521 at age 17. Nonetheless, the distribution of birth weight was similar in groups at each age. In addition, loss to follow-up bias is unlikely to influence the MR estimates, as genetic associations are unlikely to be biased by missing data [72]. This is supported by the fact that the allele scores used in this study were not strongly associated with a range of risk factors associated with loss to follow-up.

In this study we have looked at outcomes at each time point separately in order to explore whether magnitudes are similar at each age. It might also be valuable to examine whether maternal exposures relate to the rate of change in offspring adiposity across childhood and into adulthood using multilevel models. Methods of applying genome-wide data to such models have recently been developed [73]. However, we are not aware of them being used in an MR IV framework, and we feel the approach we have adopted is relevant to our aim and produces results that are easy to interpret.

Our study examined the effect of linear (incremental) increases in maternal BMI with offspring outcomes. Although methods for assessing nonlinear effects using MR have been recently developed [74], these require very large sample sizes and we are not able to apply these to our data. Thus, we cannot rule out the possibility of, for example, a nonlinear threshold effect of extreme maternal obesity having a causal intrauterine effect on offspring adiposity. However, we did not find any clear departures from linearity for either the multivariable regression or MR analyses (S7 Fig and S8 Fig). Lastly, although the pattern of positive multivariable regression and null MR results was consistent across ages in ALSPAC, we acknowledge that it would be valuable to show further replication of our results in large independent cohorts, particularly at older ages.

Given our results, together with those from sibling comparison [60] and negative control studies [54–59,65], it seems unlikely that subtle incremental differences in maternal pre- or early-pregnancy BMI play a key role in initiating or perpetuating the obesity epidemic [11,12]. Although some negative control [22,75–77] and sibling comparisons [78,79] suggest weak positive effects of maternal pre- or early-pregnancy BMI on offspring childhood BMI, in general, those studies are smaller than the ones finding null effects and have not explored associations into adulthood [1]. Our results showed no effect up to the age of 18 in females and males in the ALSPAC cohort. This finding is important given that a lack of effect in offspring entering their reproductive life course suggests that incrementally greater maternal adiposity across the population in pregnancy is unlikely to fuel the obesity epidemic across generations, although we cannot rule out an effect of more extreme phenotypes, such as extreme obesity or gestational diabetes [1]. In addition, we have not been able to investigate the causal effect of gestational weight gain during pregnancy on offspring adiposity using MR, given the current absence of robust genetic instruments for this exposure [80].

These findings suggest that overreliance on interventions in pregnancy to reduce population obesity may not be warranted and that consensus statements [14], which direct public health interventions to all family members [81,82] and at different stages of the life course, and not just intrauterine or early life [13], are likely to be important. For example, interventions aimed at the whole population (i.e., all family members at all life course stages), such as proposals for excess tax on obesogenic foods [83], are potentially more likely to halt the obesity epidemic than a focus on maternal pre-pregnancy BMI.

## Supporting Information

S1 Appendix Supplementary methods for investigating potential collider bias and the analysis plan. (DOCX)

S1 Fig Schematic diagram showing relationships for the MR analysis and how “collider bias” might occur when adjusting for offspring genotype. (DOCX)

S2 Fig Forest plot of association between maternal BMI and offspring BMI using multivariable linear regression in ALSPAC and Generation R. (DOCX)

S3 Fig Forest plot of association between maternal BMI allele score and offspring BMI using multivariable linear regression in ALSPAC and Generation R. (DOCX)

S4 Fig Multivariable and instrument variable meta-analysis to assess the association between maternal BMI and offspring BMI in ALSPAC and Generation R, additionally adjusted for paternal education in the IV analysis. (DOCX)

S5 Fig Multivariable and instrument variable meta-analysis to assess the association between maternal BMI and offspring BMI in ALSPAC and Generation R, restricted to European ancestry. (DOCX)

S6 Fig Scatterplot of maternal genetic associations with offspring BMI (adjusted for offspring genetic variants) against maternal genetic associations with maternal BMI, with causal estimates of maternal BMI on offspring BMI estimated by MR-Egger (solid line) and IVW (dashed line) methods. (DOCX)

S7 Fig Augmented partial residual plots to evaluate departures from linearity in observational and IV models from the ALSPAC main analysis. (DOCX)

S8 Fig Augmented partial residual plots to evaluate departures from linearity in observational and IV models from the Generation R main analysis. (DOCX)

S1 Table Details of the BMI SNPs obtained from genotyping and imputation. (DOCX)

S2 Table Descriptive characteristics of included participants in ALSPAC at each age. (DOCX)

S3 Table Associations between maternal BMI and maternal BMI allele score and possible confounding factors in ALSPAC. (DOCX)

S4 Table Characteristics of maternal BMI allele score and association with maternal BMI. (DOCX)

S5 Table F-statistics for first-stage regression of IV analyses in ALSPAC and Generation R. (DOCX)

S6 Table Associations between maternal BMI and offspring BMI from ages 7 to 18 using multivariable and IV methods with a 32-SNP allele score in ALSPAC (Discovery) cohort. (DOCX)

S7 Table Associations between maternal BMI and offspring FMI from ages 10 to 18 using multivariable and IV methods with a 32-SNP allele score in the ALSPAC (Discovery) cohort. (DOCX)

S8 Table Association between maternal BMI and offspring BMI from ages 7 to 18 using multivariable and IV methods with a 97-SNP allele score. (DOCX)

S9 Table Association between maternal BMI and offspring FMI from ages 7 to 18 using multivariable and IV methods with a 97-SNP allele score. (DOCX)

S10 Table Associations between maternal BMI and maternal BMI allele score and possible confounding factors in Generation R. (DOCX)

S11 Table Simulation to investigate the direction and magnitude of bias in observational multivariable and IV regression models. (DOCX)

S12 Table Association between transmitted and non-transmitted maternal haplotype scores and offspring BMI at age 7 in ALSPAC (Total n = 3,720). (DOCX)

S13 Table Regression estimates for the application of MR-Egger and IVW methods for MR in ALSPAC and Generation R. (DOCX)

S14 Table Confounder-adjusted multivariable associations of maternal pregnancy BMI with offspring BMI and FMI from ages 7 to 18 in ALSPAC (Discovery sample). (DOCX)

## Abbreviations

ALSPAC: Avon Longitudinal Study of Parents and Children
BMI: body mass index
DXA: dual-energy X-ray absorptiometry
FMI: fat mass index
IV: instrumental variable
IVW: inverse-variance weighted
GWAS: genome-wide association studies
MR: Mendelian randomisation
SD: standard deviation
