# Supplementary material for: Using Genetic Variation to Explore the Causal Effect of Maternal Pregnancy Adiposity on Future Offspring Adiposity: A Mendelian Randomisation Study
Source: PLoS Med. 2017 Jan 24;14(1):e1002221. doi: 10.1371/journal.pmed.1002221 (PMC5261553; doi:10.1371/journal.pmed.1002221)
Supplement: S1 Appendix — (DOCX) [file pmed.1002221.s001.docx]

# Appendix

## **Supplementary methods**

### **Investigating potential collider bias**

Using DAG terminology, a variable is a collider on that path if it has at least two arrows entering it i.e. is a common effect. If there is a collider on a path it is automatically blocked and therefore conditioning on (or adjusting for) a collider in a backdoor path could open it. This can lead to a phenomenon called collider bias which may result in a (non-causal) association between the variables that affect it.[1] In this situation, offspring genotype is a collider of maternal and paternal genotype and so, by adjusting for it, a different pathway from maternal genotype to paternal genotype, and then to offspring adiposity via paternal adiposity might be created (**S5 Fig**).

We carried out simulations to investigate the direction and magnitude of collider bias. This was done by first simulating data on 32 minor allele frequencies, generating maternal and paternal genotypes assuming HWE, generating offspring genotypes based on randomly allocated alleles from the mothers and fathers. From these, allele scores composed of the 32 genotypes were generated for the mothers, fathers and offspring. Further to this, BMI for the mothers and fathers was generated as a function of the relevant allele score and a simulated confounder, with values normally distributed and roughly equivalent to those seen in the three cohorts. Offspring BMI was generated as a function of allele score, maternal and paternal BMI as well as a simulated confounder. Simulations were run with 10,000 observations and 10,000 replications to fit models for the basic multivariable regression performed in the three cohorts and both the basic and offspring genotype-adjusted IV regressions.

This provided evidence that the IV model correcting for offspring genotype is biased slightly towards the null, producing an effect estimate of 0.03 compared with the true effect in this simulation which was 0.05. However, it is the least biased of the three models examined (**S11 Table**).

Within the ALSPAC cohort, using a recently described method [2] to derive parental transmission of SNP data, we constructed a haplotype genetic score derived from non-transmitted alleles to be used as a valid genetic instrument which is less susceptible to issues of collider bias. To implement this method, we inferred maternal-offspring allelic transmission based on either direct comparison of genotypes or local haplotype sharing using pre-phased genotype data for the ALSPAC mothers and children. We constructed both 32 and 97-SNP maternal transmitted and maternal non-transmitted haplotype scores and performed regression analyses of these allele scores on offspring BMI.

### **Analysis plan**

Inclusion criteria:

- Singleton live births
- White Europeans

Exposures:

- Maternal pre-pregnancy BMI

Outcomes:

- Offspring BMI at all available ages

Genotypes:

- Maternal genotype (32 BMI SNPs)
- Offspring genotype (32 BMI SNPs)

Covariables:

- Maternal age at birth
- Current age of offspring at assessment
- Sex of offspring
- Offspring weight and height at all available ages
- Ethnicity principal components (if relevant)

Derived variables:

- Generate sex and age (in month categories)-standardized z-scores of offspring BMI
- Age (in 1-year categories)-standardised z-score for maternal BMI

Confounders :

- Parity
- Parental occupation
- Highest educational qualification
- Smoking in pregnancy
- Paternal BMI

Statistical analysis:

Observational analysis

- Linear regression:

1) age-standardized maternal BMI and age and sex-standardized offspring BMI

2) adjusted for parental social class, parental education, maternal smoking during pregnancy, parity, paternal BMI

Direct genotype associations with BMI

- Derive weighted allele score for mothers and offspring based on 32 SNPs
- Linear regression:

1) maternal allele score and age-standardized maternal BMI

2) offspring allele score and age- and sex-standardized offspring BMI

Direct genotype associations with potential confounder associations

- Linear regression:

1) maternal allele score and potential confounders

2) offspring allele score and potential confounders

Direct genotype associations of maternal allele score with offspring BMI

- Linear regression:

1) maternal allele score and age-and sex-standardized offspring BMI at all available ages

2) maternal allele score and age-and sex-standardized offspring BMI at all available ages, adjusted for offspring genotype

Instrumental variable analysis

- Two-stage least squares regression:

1) age-standardized maternal BMI, instrumented by maternal allele score, and age and sex-standardized offspring BMI

2) age-standardized maternal BMI, instrumented by maternal allele score, and age and sex-standardized offspring BMI, adjusted for offspring genotype

Meta-analysis

- Inverse-variance weighted meta-analysis.

Sensitivity analysis

- Use of 97 SNP allele score in ALSPAC
- Investigation of collider bias through simulations
- Investigation of pleiotropy using MR Egger approach
- Post hoc analyses:
  - Untransmitted haplotype approach in ALSPAC
  - Investigation of non-linearity in models
  - Adjustment for maternal and offspring allele scores in multivariable regression models in ALSPAC
  - Investigation of the causal effect of maternal adiposity on offspring ponderal index in ALSPAC

References

1. Greenland S. Quantifying biases in causal models: classical confounding vs collider-stratification bias. Epidemiology. 2003;14(3):300-6. PubMed PMID: 12859030.

2. Zhang G, Bacelis J, Lengyel C, Teramo K, Hallman M, Helgeland O, et al. Assessing the Causal Relationship of Maternal Height on Birth Size and Gestational Age at Birth: A Mendelian Randomization Analysis. Plos Med. 2015;12(8). doi: ARTN e1001865 10.1371/journal.pmed.1001865. PubMed PMID: WOS:000360708300007.
