# Supplementary material for: Using Genetic Variation to Explore the Causal Effect of Maternal Pregnancy Adiposity on Future Offspring Adiposity: A Mendelian Randomisation Study
Source: PLoS Med. 2017 Jan 24;14(1):e1002221. doi: 10.1371/journal.pmed.1002221 (PMC5261553; doi:10.1371/journal.pmed.1002221)
Supplement: S1 Fig — (DOCX) [file pmed.1002221.s002.docx]

#### Supplementary Figure 1 – Schematic diagram showing relationships for the Mendelian randomization analysis and how “collider bias” might occur when adjusting for offspring genotype

Maternal genotype

Maternal adiposity

Offspring adiposity

Offspring genotype

Paternal genotype

Paternal adiposity

Confounders

The box indicates that offspring genotype has been adjusted for. While this will remove the bias caused by the path from maternal genotype via offspring genotype to offspring adiposity, it will induce a spurious association between maternal and paternal genotype (collider bias). If paternal genotype is associated with offspring adiposity (directly or through its phenotype), then any estimate of association between maternal genotype and offspring adiposity could be biased because there would be a backdoor path from maternal genotype to paternal genotype and to offspring adiposity.
