# Supplementary material for: Using Genetic Variation to Explore the Causal Effect of Maternal Pregnancy Adiposity on Future Offspring Adiposity: A Mendelian Randomisation Study
Source: PLoS Med. 2017 Jan 24;14(1):e1002221. doi: 10.1371/journal.pmed.1002221 (PMC5261553; doi:10.1371/journal.pmed.1002221)
Supplement: S2 Fig — (DOCX) [file pmed.1002221.s003.docx]

#### Supplementary Figure 2 - Forest plot of association between maternal BMI and offspring BMI using multivariable linear regression in ALSPAC and Generation R


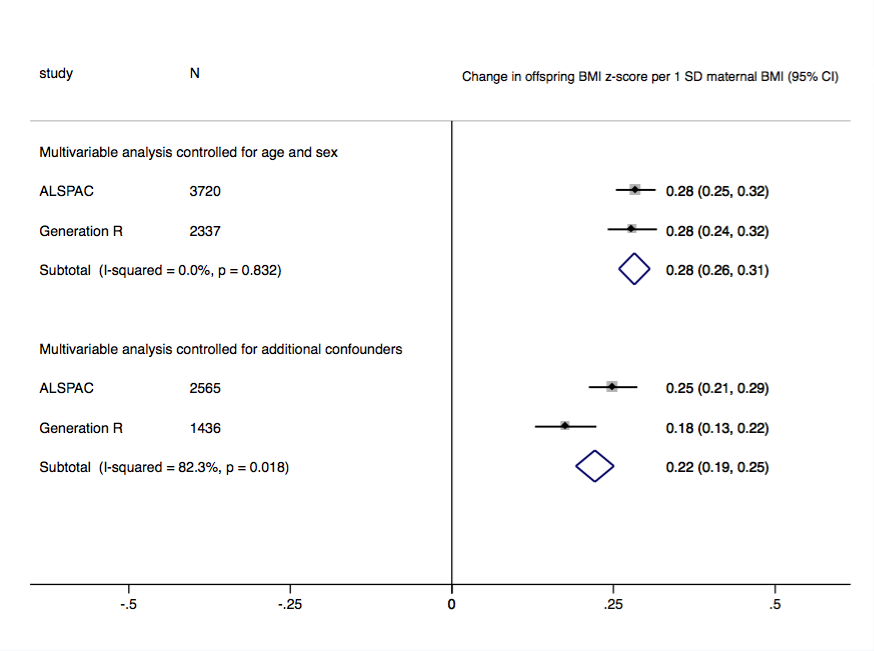


All models used age-standardised values of maternal BMI and age- and sex-standardised values of offspring BMI combined in a fixed effects meta-analysis. Multivariable analysis controlled for additional confounders adjusted for socio-economic position, parity, paternal BMI, maternal smoking during pregnancy. Additional adjustment for ethnicity was undertaking in Generation R by including the top 20 principal components obtained from PCA on offspring genome-wide data in the multivariable analyses.
