# Supplementary material for: Using Genetic Variation to Explore the Causal Effect of Maternal Pregnancy Adiposity on Future Offspring Adiposity: A Mendelian Randomisation Study
Source: PLoS Med. 2017 Jan 24;14(1):e1002221. doi: 10.1371/journal.pmed.1002221 (PMC5261553; doi:10.1371/journal.pmed.1002221)
Supplement: S4 Fig — (DOCX) [file pmed.1002221.s005.docx]

#### Supplementary Figure 4 - Multivariable and instrument variable meta-analysis to assess the association between maternal BMI and offspring BMI in ALSPAC and Generation R, additionally adjusted for paternal education in the IV analysis

* In these analyses results using the 97 BMI SNPs were used in ALSPAC and pooled with results using the 32-SNPs in Generation-R
