# Supplementary material for: Using Genetic Variation to Explore the Causal Effect of Maternal Pregnancy Adiposity on Future Offspring Adiposity: A Mendelian Randomisation Study
Source: PLoS Med. 2017 Jan 24;14(1):e1002221. doi: 10.1371/journal.pmed.1002221 (PMC5261553; doi:10.1371/journal.pmed.1002221)
Supplement: S6 Fig — (DOCX) [file pmed.1002221.s007.docx]

#### Supplementary Figure 6 – Scatter plot of maternal genetic associations with offspring BMI (adjusted for offspring genetic variants) against maternal genetic associations with maternal BMI, with causal estimates of maternal BMI on offspring BMI estimated by MR-Egger (solid line) and inverse-variance weighted (dashed line) methods.


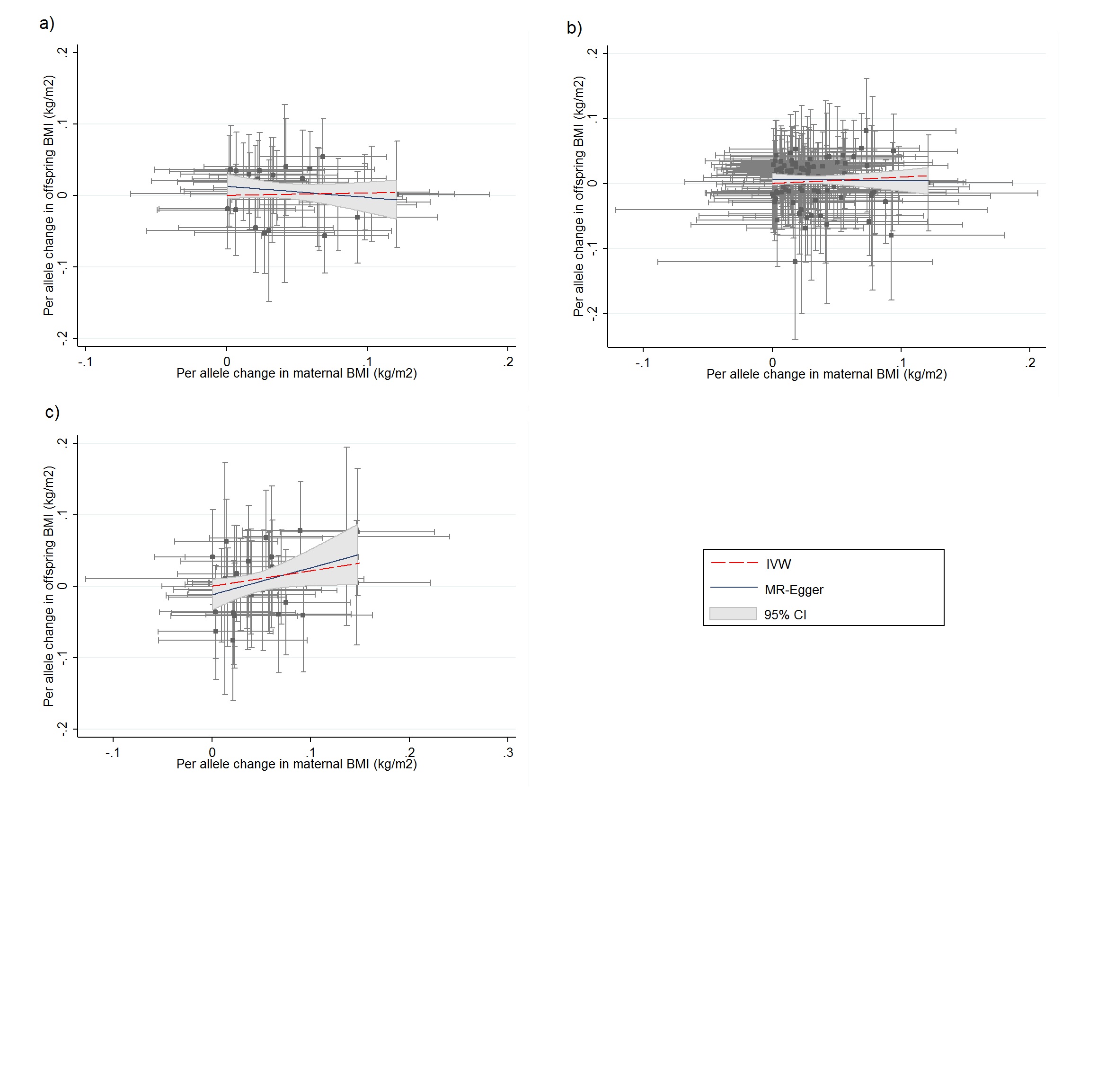


1. Causal estimates obtained using 32 SNPs in ALSPAC b) Causal estimates obtained using 97 SNPs in ALSPAC c) Causal estimates obtained using 32 SNPs in Generation R
