# Supplementary material for: Using Genetic Variation to Explore the Causal Effect of Maternal Pregnancy Adiposity on Future Offspring Adiposity: A Mendelian Randomisation Study
Source: PLoS Med. 2017 Jan 24;14(1):e1002221. doi: 10.1371/journal.pmed.1002221 (PMC5261553; doi:10.1371/journal.pmed.1002221)
Supplement: S8 Fig — (DOCX) [file pmed.1002221.s009.docx]

#### Supplementary Figure 8 – Augmented partial residual plots to evaluate departures from linearity in observational and instrumental variable models from the Generation R main analysis


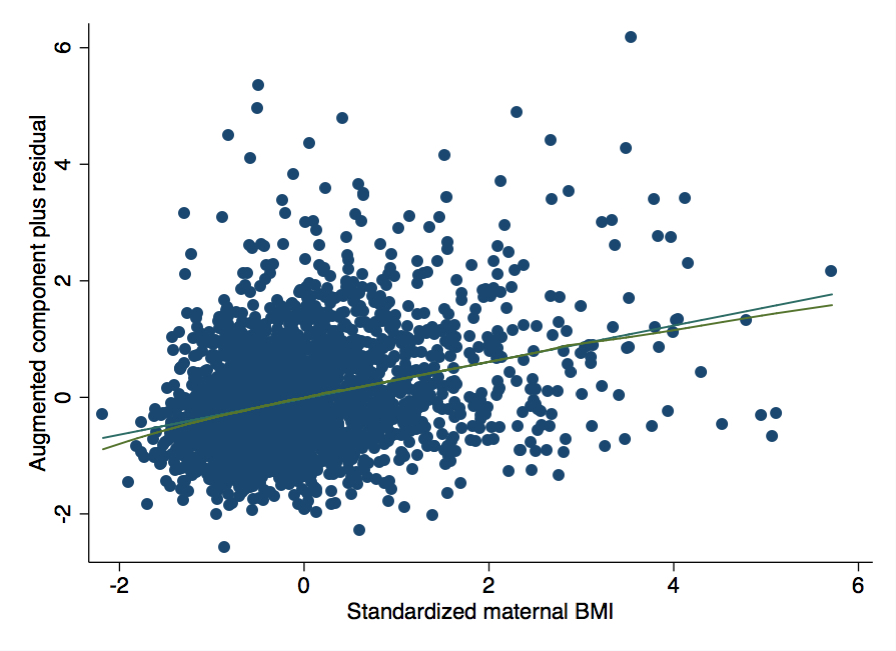


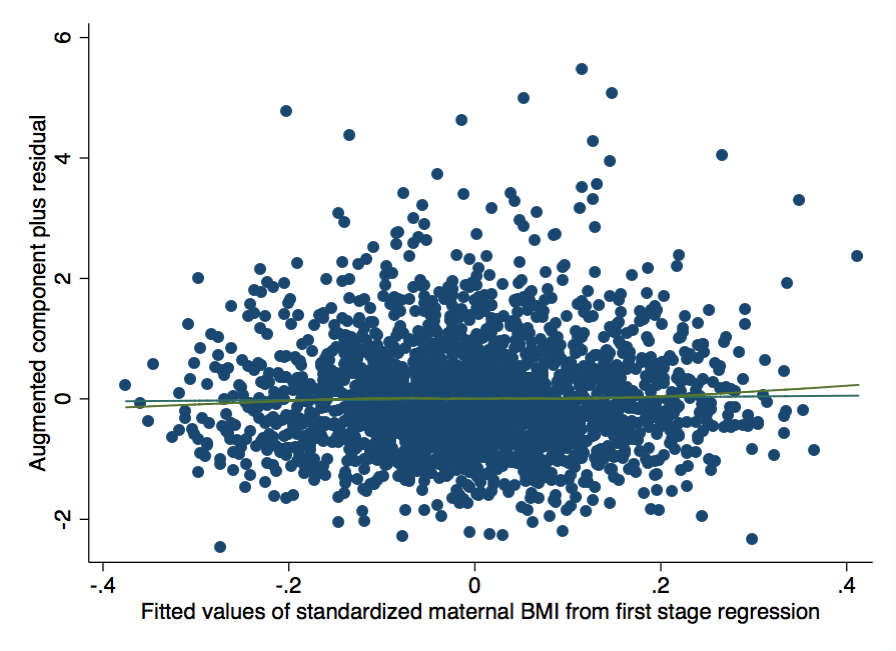


1. Augmented partial residual plot for association between standardized maternal BMI and offspring BMI in ALSPAC (observational analysis)
2. Augmented partial residual plot for association between fitted values of standardized maternal BMI on the maternal 32-SNP allele score and offspring BMI, adjusted for offspring 32-SNP allele score, in ALSPAC (instrumental variable analysis)

Dark blue line = line of best fit, Green line = non-parametric loess smoother
