# Supplementary material for: Using Genetic Variation to Explore the Causal Effect of Maternal Pregnancy Adiposity on Future Offspring Adiposity: A Mendelian Randomisation Study
Source: PLoS Med. 2017 Jan 24;14(1):e1002221. doi: 10.1371/journal.pmed.1002221 (PMC5261553; doi:10.1371/journal.pmed.1002221)
Supplement: S1 Table — (DOCX) [file pmed.1002221.s010.docx]

#### Supplementary Table 1 - Details of the BMI SNPs obtained from genotyping and imputation

| SNP | Chromosome | Base pair position* | Nearest gene | Effect allele | Other allele | EAF | GWAS | Effect size^†^ |
| --- | --- | --- | --- | --- | --- | --- | --- | --- |
| rs11583200 | 1 | 5283252 | *ELAVL4* | C | T | 40% | Locke 2015 | 0.018 |
| rs977747 | 1 | 47684677 | *TAL1* | T | G | 40% | Locke 2015 | 0.017 |
| rs657452 | 1 | 49589847 | *AGBL4* | A | G | 39% | Locke 2015 | 0.023 |
| rs3101336^‡^ | 1 | 72751185 | *NEGR1* | C | T | 61% | Locke 2015 | 0.033 |
| rs2815752^‡^ | 1 | 72812440 | *NEGR1* | A | G | 61% | Speliotes 2012 | 0.13 |
| rs1514175^‡^ | 1 | 74991644 | *TNNI3K* | A | G | 43% | Speliotes 2012 | 0.07 |
| rs12566985^‡^ | 1 | 75002193 | *FPGT-TNNI3K* | G | A | 45% | Locke 2015 | 0.024 |
| rs12401738 | 1 | 78446761 | *FUBP1* | A | G | 35% | Locke 2015 | 0.021 |
| rs11165643^‡^ | 1 | 96924097 | *PTBP2* | T | C | 58% | Locke 2015 | 0.022 |
| rs1555543^‡^ | 1 | 96944797 | *PTBP2* | C | A | 59% | Speliotes 2012 | 0.06 |
| rs543874 | 1 | 177889480 | *SEC16B* | G | A | 19% | Speliotes 2012  Locke 2015 | 0.22  0.048 |
| rs2820292 | 1 | 201784287 | *NAV1* | C | A | 56% | Locke 2015 | 0.02 |
| rs17024393 | 1 | 110154688 | *GNAT2* | C | T | 4% | Locke 2015 | 0.066 |
| rs2867125^‡^ | 2 | 622827 | *TMEM18* | C | T | 83% | Speliotes 2012 | 0.31 |
| rs13021737^‡^ | 2 | 632348 | *TMEM18* | G | A | 83% | Locke 2015 | 0.06 |
| rs10182181^‡^ | 2 | 25150296 | *ADCY3* | G | A | 46% | Locke 2015 | 0.031 |
| rs713586^‡^ | 2 | 25158008 | *RBJ* | C | T | 47% | Speliotes 2012 | 0.14 |
| rs11126666 | 2 | 26928811 | *KCNK3* | A | G | 28% | Locke 2015 | 0.021 |
| rs887912^‡^ | 2 | 59302877 | *FANCL* | T | C | 29% | Speliotes 2012 | 0.10 |
| rs1016287^‡^ | 2 | 59305625 | *FLJ30838* | T | C | 29% | Locke 2015 | 0.023 |
| rs11688816 | 2 | 63053048 | *EHBP1* | G | A | 53% | Locke 2015 | 0.017 |
| rs2890652 | 2 | 142959931 | *LRP1B* | C | T | 18% | Speliotes 2012 | 0.09 |
| rs2121279 | 2 | 143043285 | *LRP1B* | T | C | 15% | Locke 2015 | 0.025 |
| rs1460676 | 2 | 164567689 | *FIGN* | C | T | 18% | Locke 2015 | 0.021 |
| rs1528435 | 2 | 181550962 | *UBE2E3* | T | C | 63% | Locke 2015 | 0.018 |
| rs17203016 | 2 | 208255518 | *CREB1* | G | A | 20% | Locke 2015 | 0.021 |
| rs7599312 | 2 | 213413231 | *ERBB4* | G | A | 72% | Locke 2015 | 0.022 |
| rs492400 | 2 | 219349752 | *USP37* | C | T | 42% | Locke 2015 | 0.024 |
| rs2176040 | 2 | 227092802 | *LOC646736* | A | G | 37% | Locke 2015 | 0.024 |
| rs6804842 | 3 | 25106437 | *RARB* | G | A | 58% | Locke 2015 | 0.019 |
| rs2365389 | 3 | 61236462 | *FHIT* | C | T | 58% | Locke 2015 | 0.02 |
| rs3849570 | 3 | 81792112 | *GBE1* | A | C | 36% | Locke 2015 | 0.019 |
| rs13078960^‡^ | 3 | 85807590 | *CADM2* | G | T | 20% | Locke 2015 | 0.03 |
| rs13078807^‡^ | 3 | 85884150 | *CADM2* | G | A | 20% | Speliotes 2012 | 0.10 |
| rs16851483 | 3 | 141275436 | *RASA2* | T | G | 7% | Locke 2015 | 0.048 |
| rs1516725 | 3 | 185824004 | *ETV5* | C | T | 87% | Locke 2015 | 0.045 |
| rs9816226 | 3 | 185834499 | *ETV5* | T | A | 82% | Speliotes 2012 | 0.14 |
| rs10938397 | 4 | 45182527 | *GNPDA2* | G | A | 43% | Speliotes 2012 Locke 2015 | 0.18  0.04 |
| rs17001654 | 4 | 77129568 | *SCARB2* | G | C | 15% | Locke 2015 | 0.031 |
| rs13107325 | 4 | 103188709 | *SLC39A8* | T | C | 7% | Speliotes 2012 Locke 2015 | 0.19  0.048 |
| rs11727676 | 4 | 145659064 | *HHIP* | T | C | 91% | Locke 2015 | 0.036 |
| rs2112347 | 5 | 75015242 | *FLJ35779* | T | G | 63% | Speliotes 2012  Locke 2015 | 0.10  0.026 |
| rs4836133 | 5 | 124332103 | *ZNF608* | A | C | 48% | Speliotes 2012 | 0.07 |
| rs7715256 | 5 | 153537893 | *GALNT10* | G | T | 42% | Locke 2015 | 0.017 |
| rs206936 | 6 | 34302869 | *NUDT3* | G | A | 21% | Speliotes 2012 | 0.06 |
| rs205262 | 6 | 34563164 | *C6orf106* | G | A | 27% | Locke 2015 | 0.022 |
| rs2033529 | 6 | 40348653 | *TDRG1* | G | A | 29% | Locke 2015 | 0.019 |
| rs987237^‡^ | 6 | 50803050 | *TFAP2B* | G | A | 18% | Speliotes 2012 | 0.13 |
| rs2207139^‡^ | 6 | 50845490 | *TFAP2B* | G | A | 18% | Locke 2015 | 0.045 |
| rs9400239 | 6 | 108977663 | *FOXO3* | C | T | 69% | Locke 2015 | 0.019 |
| rs9374842 | 6 | 120185665 | *LOC285762* | T | C | 74% | Locke 2015 | 0.023 |
| rs13201877 | 6 | 137675541 | *IFNGR1* | G | A | 14% | Locke 2015 | 0.024 |
| rs13191362 | 6 | 163033350 | *PARK2* | A | G | 88% | Locke 2015 | 0.028 |
| rs1167827 | 7 | 75163169 | *HIP1* | G | A | 55% | Locke 2015 | 0.02 |
| rs2245368 | 7 | 76608143 | *PMS2L11* | C | T | 18% | Locke 2015 | 0.032 |
| rs9641123 | 7 | 93197732 | *CALCR* | C | G | 43% | Locke 2015 | 0.029 |
| rs6465468 | 7 | 95169514 | *ASB4* | T | G | 31% | Locke 2015 | 0.025 |
| rs17405819 | 8 | 76806584 | *HNF4G* | T | C | 70% | Locke 2015 | 0.022 |
| rs16907751 | 8 | 81375457 | *ZBTB10* | C | T | 91% | Locke 2015 | 0.047 |
| rs2033732 | 8 | 85079709 | *RALYL* | C | T | 75% | Locke 2015 | 0.019 |
| rs4740619 | 9 | 15634326 | *C9orf93* | T | C | 54% | Locke 2015 | 0.018 |
| rs10968576 | 9 | 28414339 | *LRRN6C* | G | A | 32% | Speliotes 2012  Locke 2015 | 0.11  0.025 |
| rs6477694 | 9 | 111932342 | *EPB41L4B* | C | T | 37% | Locke 2015 | 0.017 |
| rs1928295 | 9 | 120378483 | *TLR4* | T | C | 55% | Locke 2015 | 0.019 |
| rs10733682 | 9 | 129460914 | *LMX1B* | A | G | 48% | Locke 2015 | 0.017 |
| rs7899106 | 10 | 87410904 | *GRID1* | G | A | 5% | Locke 2015 | 0.04 |
| rs17094222 | 10 | 102395440 | *HIF1AN* | C | T | 21% | Locke 2015 | 0.025 |
| rs11191560 | 10 | 104869038 | *NT5C2* | C | T | 9% | Locke 2015 | 0.031 |
| rs7903146 | 10 | 114758349 | *TCF7L2* | C | T | 71% | Locke 2015 | 0.023 |
| rs4929949 | 11 | 8604593 | *RPL27A* | C | T | 52% | Speliotes 2012 | 0.06 |
| rs4256980 | 11 | 8673939 | *TRIM66* | G | C | 65% | Locke 2015 | 0.021 |
| rs11030104^‡^ | 11 | 27684517 | *BDNF* | A | G | 79% | Locke 2015 | 0.041 |
| rs10767664^‡^ | 11 | 27725986 | *BDNF* | A | T | 78% | Speliotes 2012 | 0.19 |
| rs2176598 | 11 | 43864278 | *HSD17B12* | T | C | 25% | Locke 2015 | 0.02 |
| rs3817334 | 11 | 47650993 | *MTCH2* | T | C | 41% | Speliotes 2012 Locke 2015 | 0.06  0.026 |
| rs12286929 | 11 | 115022404 | *CADM1* | G | A | 52% | Locke 2015 | 0.022 |
| rs7138803 | 12 | 50247468 | *FAIM2* | A | G | 38% | Speliotes 2012 Locke 2015 | 0.12  0.032 |
| rs11057405 | 12 | 122781897 | *CLIP1* | G | A | 90% | Locke 2015 | 0.031 |
| rs12016871^‡^ | 13 | 28017782 | *MTIF3* | T | C | 20% | Locke 2015 | 0.03 |
| rs4771122^‡^ | 13 | 28020180 | *MTIF3* | G | A | 24% | Speliotes 2012 | 0.09 |
| rs12429545 | 13 | 54102206 | *OLFM4* | A | G | 13% | Locke 2015 | 0.033 |
| rs9540493 | 13 | 66205704 | *MIR548X2* | A | G | 45% | Locke 2015 | 0.021 |
| rs1441264 | 13 | 79580919 | *MIR548A2* | A | G | 61% | Locke 2015 | 0.017 |
| rs10132280 | 14 | 25928179 | *STXBP6* | C | A | 68% | Locke 2015 | 0.023 |
| rs12885454 | 14 | 29736838 | *PRKD1* | C | A | 64% | Locke 2015 | 0.021 |
| rs11847697 | 14 | 30515112 | *PRKD1* | T | C | 4% | Speliotes 2012 Locke 2015 | 0.17  0.049 |
| rs7141420^‡^ | 14 | 79899454 | *NRXN3* | T | C | 53% | Locke 2015 | 0.024 |
| rs10150332^‡^ | 14 | 79936964 | *NRXN3* | C | T | 21% | Speliotes 2012 | 0.13 |
| rs3736485 | 15 | 51748610 | *DMXL2* | A | G | 45% | Locke 2015 | 0.018 |
| rs16951275^‡^ | 15 | 68077168 | *MAP2K5* | T | C | 78% | Locke 2015 | 0.031 |
| rs2241423^‡^ | 15 | 68086838 | *MAP2K5* | G | A | 78% | Speliotes 2012 | 0.13 |
| rs7164727 | 15 | 73093991 | *LOC100287559* | T | C | 67% | Locke 2015 | 0.019 |
| rs758747 | 16 | 3627358 | *NLRC3* | T | C | 27% | Locke 2015 | 0.023 |
| rs12444979^‡^ | 16 | 19933600 | *GPRC5B* | C | T | 87% | Speliotes 2012 | 0.17 |
| rs12446632^‡^ | 16 | 19935389 | *GPRC5B* | G | A | 87% | Locke 2015 | 0.04 |
| rs2650492 | 16 | 28333411 | *SBK1* | A | G | 30% | Locke 2015 | 0.021 |
| rs7359397^‡^ | 16 | 28885659 | *SH2B1* | T | C | 40% | Speliotes 2012 | 0.15 |
| rs3888190^‡^ | 16 | 28889486 | *ATP2A1* | A | C | 40% | Locke 2015 | 0.031 |
| rs4787491 | 16 | 30015337 | *INO80E* | G | A | 51% | Locke 2015 | 0.022 |
| rs9925964 | 16 | 31129895 | *KAT8* | A | G | 62% | Locke 2015 | 0.019 |
| rs2080454 | 16 | 49062590 | *CBLN1* | C | A | 41% | Locke 2015 | 0.017 |
| rs1558902 | 16 | 53803574 | *FTO* | A | T | 42% | Speliotes 2012  Locke 2015 | 0.39  0.082 |
| rs9914578 | 17 | 2005136 | *SMG6* | G | C | 23% | Locke 2015 | 0.02 |
| rs1000940 | 17 | 5283252 | *RABEP1* | G | A | 32% | Locke 2015 | 0.019 |
| rs12940622 | 17 | 78615571 | *RPTOR* | G | A | 58% | Locke 2015 | 0.018 |
| rs1808579 | 18 | 21104888 | *C18orf8* | C | T | 53% | Locke 2015 | 0.017 |
| rs7239883 | 18 | 40147671 | *LOC284260* | G | A | 39% | Locke 2015 | 0.023 |
| rs7243357 | 18 | 56883319 | *GRP* | T | G | 81% | Locke 2015 | 0.022 |
| rs6567160^‡^ | 18 | 57829135 | *MC4R* | C | T | 24% | Locke 2015 | 0.056 |
| rs571312^‡^ | 18 | 57839769 | *MC4R* | A | C | 24% | Speliotes 2012 | 0.23 |
| rs17724992 | 19 | 18454825 | *PGPEP1* | A | G | 75% | Locke 2015 | 0.019 |
| rs29941 | 19 | 34309532 | *KCTD15* | G | A | 67% | Speliotes 2012 Locke 2015 | 0.06  0.018 |
| rs2075650 | 19 | 45395619 | *TOMM40* | A | G | 85% | Locke 2015 | 0.026 |
| rs2287019 | 19 | 46202172 | *QPCTL* | C | T | 80% | Speliotes 2012 Locke 2015 | 0.15  0.036 |
| rs3810291 | 19 | 47569003 | *TMEM160* | A | G | 67% | Speliotes 2012 Locke 2015 | 0.09  0.028 |
| rs6091540 | 20 | 51087862 | *ZFP64* | C | T | 72% | Locke 2015 | 0.03 |
| rs2836754 | 21 | 40291740 | *ETS2* | C | T | 60% | Locke 2015 | 0.017 |

EAF = effect allele frequency taken from GWAS in which SNP was identified, GWAS = Genome-wide association study in which SNP was identified *Base pair position based on Genome Reference Consortium GRCh37 ^†^Effect size taken from GWAS in which SNP was identified ^‡^Linkage disequilibrium r2 > 0.8 from SNP Annotation and Proxy Search tool, SNAP
