# Supplementary material for: Using Genetic Variation to Explore the Causal Effect of Maternal Pregnancy Adiposity on Future Offspring Adiposity: A Mendelian Randomisation Study
Source: PLoS Med. 2017 Jan 24;14(1):e1002221. doi: 10.1371/journal.pmed.1002221 (PMC5261553; doi:10.1371/journal.pmed.1002221)
Supplement: S2 Table — (DOCX) [file pmed.1002221.s011.docx]

#### Supplementary Table 2 - Descriptive characteristics of included participants in ALSPAC at each age

|  | Age 7 | Age 10 | Age 12 | Age 14 | Age 16 | Age 18 |
| --- | --- | --- | --- | --- | --- | --- |
| N* | 3,720 | 3,657 | 3,496 | 3,227 | 2,806 | 2,521 |
| Males | 48.6% | 48.0% | 47.7% | 47.6% | 46.4% | 43.6% |
| Age (months) | 89.6 (1.9) | 118.1 (3.5) | 140.7 (2.7) | 166.0 (2.4) | 185.1 (3.5) | 213.3 (4.8) |
| N | 3,673 | 3,612 | 3,452 | 3,185 | 2,768 | 2,491 |
| Birth weight (g) | 3464.8 (510.5) | 3451.5 (515.4) | 3454.8  (515.9) | 3448.9  (516.2) | 3448.9 (514.5) | 3448.2 (513.2) |
| Weight (kg) | 25.6 (4.4) | 34.5 (7.2) | 43.4 (9.8) | 54.2 (10.9) | 61.0 (11.4) | 66.5 (13.4) |
| Height (cm) | 16.2 (2.0) | 139.6 (6.3) | 150.8 (7.3) | 163.5 (7.8) | 169.3 (8.4) | 171.4 (9.4) |
| BMI (kg/m^2^) | 22.9 (3.7) | 17.6 (2.8) | 18.9 (3.3) | 20.2 (3.4) | 21.2 (3.3) | 22.6 (3.9) |
| N | - | 3,495 | 3,444 | 3,192 | 2,715 | 2,430 |
| Fat mass (kg) | - | 8.4 (4.9) | 11.5 (6.5) | 13.5 (7.8) | 15.1 (8.8) | 17.8 (9.9) |
| Lean mass (kg) | - | 24.5 (3.2) | 29.6 (4.3) | 37.9 (6.4) | 42.9 (8.3) | 45.6 (10.0) |
| FMI (kg/m^2^) | - | 4.3 (2.3) | 5.0 (2.6) | 5.1 (2.9) | 5.3 (3.1) | 6.2 (3.5) |
| Maternal BMI (kg/m^2^) | 22.9 (3.7) | 22.9 (3.7) | 22.9 (3.7) | 22.8 (3.6) | 22.8 (3.6) | 22.8 (3.7) |

*Total N unless otherwise specified
