# Supplementary material for: Using Genetic Variation to Explore the Causal Effect of Maternal Pregnancy Adiposity on Future Offspring Adiposity: A Mendelian Randomisation Study
Source: PLoS Med. 2017 Jan 24;14(1):e1002221. doi: 10.1371/journal.pmed.1002221 (PMC5261553; doi:10.1371/journal.pmed.1002221)
Supplement: S3 Table — (DOCX) [file pmed.1002221.s012.docx]

#### Supplementary Table 3 - Associations between maternal BMI and maternal BMI allele score and possible confounding factors in ALSPAC

| Variable (categories/units) | N* | Association of maternal body mass index with confounders | | Association of maternal BMI allele score with confounders | |
| --- | --- | --- | --- | --- | --- |
|  |  | Coefficient^†^  (95% CI) | P-value | Coefficient^†^  (95% CI) | P-value |
| Parental social class (I, II, III(NM), III(M), IV, V) | 3,548 | 0.11  (0.08, 0.14) | 5.04 x 10^-11^ | 0.02  (-0.01, 0.05) | 0.27 |
| Maternal education (CSE/vocational, O-level, A-level, Degree) | 3,574 | -0.10  (-0.14, -0.07) | 8.79 x 10^-10^ | -0.02  (-0.05, 0.02) | 0.36 |
| Paternal education (CSE/vocational, O-level, A-level, Degree) | 3,423 | -0.10  (-0.13, -0.07) | 5.91 x 10^-11^ | -0.03  (-0.07, 0.00) | 0.03 |
| Maternal smoking in pregnancy (none, early, throughout pregnancy) | 3,564 | 0.02  (-0.03, 0.06) | 0.48 | 0.04  (-0.01, 0.08) | 0.12 |
| Maternal parity (0, 1, 2, 3+) | 3,654 | 0.04  (0.01, 0.08) | 0.02 | 0.02  (-0.02, 0.06) | 0.26 |
| Paternal BMI (kg/m^2^) | 2,822 | 0.06  (0.05, 0.07) | 2.33 x 10^-23^ | 0.00  (-0.01, 0.02) | 0.55 |

*N based on full sample with offspring BMI at age 7

^†^Effect estimates are in terms of age-standardised maternal BMI and standardised allele scores per unit or category increase of the confounder
