# Supplementary material for: Using Genetic Variation to Explore the Causal Effect of Maternal Pregnancy Adiposity on Future Offspring Adiposity: A Mendelian Randomisation Study
Source: PLoS Med. 2017 Jan 24;14(1):e1002221. doi: 10.1371/journal.pmed.1002221 (PMC5261553; doi:10.1371/journal.pmed.1002221)
Supplement: S4 Table — (DOCX) [file pmed.1002221.s013.docx]

#### Supplementary Table 4 – Characteristics of maternal BMI allele score and association with maternal BMI

|  |  |  |  | Association of maternal allele score with maternal pre-pregnancy BMI | | |
| --- | --- | --- | --- | --- | --- | --- |
| Study | Allele score | Mean number of BMI-increasing alleles | SD | Coefficient (SE)* | P-value | R^2^ |
| ALSPAC (N=3,720) | 32-SNP score | 29.2 | 3.9 | 0.037 (0.004) | 1.57 x 10^-19^ | 0.022 |
| ALSPAC (N=3,720) | 97-SNP score | 88.6 | 6.1 | 0.026 (0.003) | 4.60 x 10^-23^ | 0.026 |
| Generation R^†^ (N=2,337) | 32 SNP-score | 28.4 | 3.9 | 0.035 (0.005) | 2.56 x 10^-11^ | 0.019 |
| Generation R (Europeans only) (N=1.280) | 32 SNP-score | 29.0 | 3.9 | 0.032 (0.007) | 8.59 x 10^-6^ | 0.015 |

*Coefficients are displayed as age-specific z-scores per allele increase in weighted allele score

^†^Ethnicity was adjusted for using first 20 genomic components from the offspring GWAS PCA
