# Supplementary material for: Using Genetic Variation to Explore the Causal Effect of Maternal Pregnancy Adiposity on Future Offspring Adiposity: A Mendelian Randomisation Study
Source: PLoS Med. 2017 Jan 24;14(1):e1002221. doi: 10.1371/journal.pmed.1002221 (PMC5261553; doi:10.1371/journal.pmed.1002221)
Supplement: S5 Table — (DOCX) [file pmed.1002221.s014.docx]

#### Supplementary Table 5 – F-statistics for first stage regression of instrumental variable analyses in ALSPAC and Generation R

| Analysis set | Number of SNPs in allele score | F-statistic without offspring allele score adjustment | F-statistic with offspring allele score adjustment |
| --- | --- | --- | --- |
| ALSPAC offspring BMI age 7 | 32 | 82 | 59 |
| ALSPAC offspring BMI age 10 | 32 | 82 | 57 |
| ALSPAC offspring BMI age 12 | 32 | 73 | 58 |
| ALSPAC offspring BMI age 14 | 32 | 70 | 57 |
| ALSPAC offspring BMI age 16 | 32 | 58 | 48 |
| ALSPAC offspring FMI age 18 | 32 | 58 | 48 |
| ALSPAC offspring FMI age 10 | 32 | 73 | 53 |
| ALSPAC offspring FMI age 12 | 32 | 74 | 58 |
| ALSPAC offspring FMI age 14 | 32 | 70 | 58 |
| ALSPAC offspring FMI age 16 | 32 | 58 | 47 |
| ALSPAC offspring FMI age 18 | 32 | 54 | 45 |
| ALSPAC offspring BMI age 7 | 97 | 82 | 59 |
| ALSPAC offspring BMI age 10 | 97 | 110 | 73 |
| ALSPAC offspring BMI age 12 | 97 | 95 | 71 |
| ALSPAC offspring BMI age 14 | 97 | 90 | 74 |
| ALSPAC offspring BMI age 16 | 97 | 83 | 68 |
| ALSPAC offspring FMI age 18 | 97 | 76 | 67 |
| ALSPAC offspring FMI age 10 | 97 | 105 | 72 |
| ALSPAC offspring FMI age 12 | 97 | 96 | 71 |
| ALSPAC offspring FMI age 14 | 97 | 88 | 74 |
| ALSPAC offspring FMI age 16 | 97 | 85 | 69 |
| ALSPAC offspring FMI age 18 | 97 | 72 | 63 |
| Generation R offspring BMI age 6 | 32 | 44 | 30 |
