# Supplementary material for: Using Genetic Variation to Explore the Causal Effect of Maternal Pregnancy Adiposity on Future Offspring Adiposity: A Mendelian Randomisation Study
Source: PLoS Med. 2017 Jan 24;14(1):e1002221. doi: 10.1371/journal.pmed.1002221 (PMC5261553; doi:10.1371/journal.pmed.1002221)
Supplement: S6 Table — (DOCX) [file pmed.1002221.s015.docx]

#### Supplementary Table 6 - Associations between maternal BMI and offspring BMI from age 7 to 18 using multivariable and instrumental variable methods with a 32-SNP allele score in ALSPAC (discovery) cohort

| Difference in mean offspring BMI (SD) per 1SD maternal pre-pregnancy BMI from multivariable regression. | | | | | | | Difference in mean offspring BMI (SD) per 1SD maternal pre-pregnancy BMI from genetic instrumental variable (Mendelian randomization) analyses. | | | | | | |
| --- | --- | --- | --- | --- | --- | --- | --- | --- | --- | --- | --- | --- | --- |
|  | Model 1 | | | Model 2 | | | Model 3 | | | Model 4 | | | |
| Outcome | N | Result  (95% CI) | P-value | N | Result  (95% CI) | P-value | N | Result (95% CI) | P-value | N | Result  (95% CI) | P-value | p(diff)b |
| BMI  age 7 | 3,720 | 0.28  (0.25, 0.32) | 1.85 x 10^-73^ | 2,565 | 0.25  (0.21, 0.29) | 9.42 x10^-39^ | 3,720 | 0.51  (0.30, 0.73) | 3.16 x10^-6^ | 3,720 | 0.04  (-0.21, 0.30) | 0.73 | 0.13 |
| BMI  age 10 | 3,657 | 0.33  (0.30, 0.36) | 2.44 x 10^-95^ | 2,507 | 0.31  (0.27, 0.35) | 6.21 x10^-60^ | 3,657 | 0.65  (0.43, 0.87) | 1.07 x10^-8^ | 3,657 | 0.03  (-0.23, 0.29) | 0.83 | 0.03 |
| BMI  age 12 | 3,496 | 0.35  (0.32, 0.38) | 7.89 x 10^-103^ | 2,411 | 0.32  (0.29, 0.36) | 2.81 x10^-63^ | 3,496 | 0.64  (0.40, 0.87) | 7.45 x10^-8^ | 3,496 | 0.00  (-0.26, 0.26) | 0.99 | 0.02 |
| BMI  age 14 | 3,227 | 0.34  (0.31, 0.38) | 8.27 x 10^-91^ | 2,258 | 0.32  (0.28, 0.36) | 2.40 x10^-58^ | 3,227 | 0.59  (0.35, 0.82) | 1.17 x10^-6^ | 3,227 | -0.07  (-0.34, 0.20) | 0.62 | 0.01 |
| BMI  age 16 | 2,806 | 0.38  (0.34, 0.41) | 9.64 x 10^-94^ | 1,979 | 0.34  (0.30, 0.39) | 6.22 x10^-56^ | 2,806 | 0.52  (0.27, 0.78) | 5.58 x10^-5^ | 2,806 | -0.10  (-0.41, 0.20) | 0.52 | 0.003 |
| BMI  age 18 | 2,521 | 0.35  (0.31, 0.39) | 2.76 x 10^-76^ | 1,798 | 0.33  (0.28, 0.37) | 2.48 x10^-47^ | 2,521 | 0.49  (0.24, 0.74) | 1.45 x10^-4^ | 2,521 | -0.03  (-0.32, 0.26) | 0.83 | 0.01 |

All results are the difference in mean offspring BMI in standard deviation (SD) units per greater SD of maternal pregnancy BMI. Model 1 multivariable regression with control for maternal age and offspring age and sex through standardisation of maternal BMI and offspring BMI; Model 2 multivariable regression additionally adjusted for parental social class, maternal and paternal education, parity and paternal BMI; Model 3: genetic instrumental variable (Mendelian randomization) with control for maternal age and offspring age and sex through standardisation of BMI; Model 4 genetic instrumental variable (Mendelian randomization) additionally adjusted for offspring allele score.
