# Supplementary material for: Using Genetic Variation to Explore the Causal Effect of Maternal Pregnancy Adiposity on Future Offspring Adiposity: A Mendelian Randomisation Study
Source: PLoS Med. 2017 Jan 24;14(1):e1002221. doi: 10.1371/journal.pmed.1002221 (PMC5261553; doi:10.1371/journal.pmed.1002221)
Supplement: S8 Table — (DOCX) [file pmed.1002221.s017.docx]

|  | Model 1 | | | Model 2^†^ | | | Model 3 | | | Model 4^‡^ | | | |
| --- | --- | --- | --- | --- | --- | --- | --- | --- | --- | --- | --- | --- | --- |
| Outcome | N* | Z-score Coefficient^$^  (95% CI) | P-value | N | Z-score Coefficient^$^  (95% CI) | P-value | N | Z-score Coefficient^$^ (95% CI) | P-value | N | Z-score Coefficient^$^  (95% CI) | P-value | p(diff)♮ |
| BMI  age 7 | 3,720 | 0.28  (0.25, 0.32) | 1.85 x 10^-73^ | 2,565 | 0.25  (0.21, 0.29) | 9.42 x 10^-39^ | 3,720 | 0.50  (0.30, 0.73) | 3.16 x 10^-6^ | 3,720 | 0.04  (-0.21, 0.30) | 0.73 | 0.035 |
| BMI  age 10 | 3,657 | 0.33  (0.30, 0.36) | 2.44 x 10^-95^ | 2,507 | 0.31  (0.27, 0.35) | 6.21 x 10^-60^ | 3,657 | 0.69  (0.50, 0.89) | 2.95 x 10^-12^ | 3,657 | 0.07  (-0.16, 0.29) | 0.56 | 0.028 |
| BMI  age 12 | 3,496 | 0.35  (0.32, 0.38) | 7.89 x 10^-103^ | 2,411 | 0.32  (0.29, 0.36) | 2.81 x 10^-63^ | 3,496 | 0.73  (0.52, 0.94) | 9.10 x 10^-12^ | 3,496 | 0.04  (-0.20, 0.27) | 0.27 | 0.014 |
| BMI  age 14 | 3,227 | 0.34  (0.31, 0.38) | 8.27 x 10^-91^ | 2,258 | 0.32  (0.28, 0.36) | 2.40 x 10^-58^ | 3,227 | 0.67  (0.46, 0.88) | 8.79 x 10^-10^ | 3,227 | 0.06  (-0.17, 0.29) | 0.60 | 0.028 |
| BMI  age 16 | 2,806 | 0.38  (0.34, 0.41) | 9.64 x 10^-94^ | 1,979 | 0.34  (0.30, 0.39) | 6.22 x 10^-56^ | 2,806 | 0.59  (0.38, 0.81) | 7.87 x 10^-8^ | 2,806 | 0.00  (-0.25, 0.24) | 0.97 | 0.002 |
| BMI  age 18 | 2,521 | 0.35  (0.31, 0.39) | 2.76 x 10^-76^ | 1,798 | 0.33  (0.28, 0.37) | 2.48 x 10^-47^ | 2,521 | 0.59  (0.37, 0.81) | 2.47 x 10^-7^ | 2,521 | 0.02  (-0.22, 0.26) | 0.85 | 0.010 |

#### Supplementary Table 8 –Association between maternal BMI and offspring BMI from age 7 to 18 using multivariable and instrumental variable methods with a 97-SNP allele score

All results are the difference in mean offspring BMI in standard deviation (SD) units per greater SD of maternal pregnancy BMI. Model 1 multivariable regression with control for maternal age and offspring age and sex through standardisation of maternal BMI and offspring BMI; Model 2 multivariable regression additionally adjusted for parental social class, maternal and paternal education, parity and paternal BMI; Model 3: genetic instrumental variable (Mendelian randomization) with control for maternal age and offspring age and sex through standardisation of BMI; Model 4 genetic instrumental variable (Mendelian randomization) additionally adjusted for offspring allele score.
