# Supplementary material for: Using Genetic Variation to Explore the Causal Effect of Maternal Pregnancy Adiposity on Future Offspring Adiposity: A Mendelian Randomisation Study
Source: PLoS Med. 2017 Jan 24;14(1):e1002221. doi: 10.1371/journal.pmed.1002221 (PMC5261553; doi:10.1371/journal.pmed.1002221)
Supplement: S9 Table — (DOCX) [file pmed.1002221.s018.docx]

#### Supplementary Table 9 – Association between maternal BMI and offspring FMI from age 7 to 18 using multivariable and instrumental variable methods with a 97-SNP allele score

| Multivariable regression of offspring FMI on maternal BMI | | | | | | | Instrumental variable regression of offspring FMI on maternal BMI | | | | | | |
| --- | --- | --- | --- | --- | --- | --- | --- | --- | --- | --- | --- | --- | --- |
|  | Model 1 | | | Model 2^†^ | | | Model 3 | | | Model 4^‡^ | | | |
| Outcome | N* | Z-score Coefficient^$^  (95% CI) | P-value | N | Z-score Coefficient^$^  (95% CI) | P-value | N | Z-score Coefficient^$^ (95% CI) | P-value | N | Z-score Coefficient^$^  (95% CI) | P-value | p(diff)♮ |
| FMI age 10 | 3,495 | 0.31  (0.28, 0.35) | 2.88 x 10^-83^ | 2,413 | 0.30  (0.26, 0.33) | 2.07 x 10^-51^ | 3,495 | 0.69  (0.49, 0.89) | 1.82 x 10^-11^ | 3,495 | 0.10  (-0.13, 0.36) | 0.38 | 0.079 |
| FMI age 12 | 3,444 | 0.33  (0.30, 0.36) | 1.89 x 10^-87^ | 2,375 | 0.31  (0.27, 0.35) | 3.40 x 10^-56^ | 3,444 | 0.69  (0.48, 0.90) | 1.20 x 10^-10^ | 3,444 | 0.06  (-0.18, 0.30) | 0.63 | 0.033 |
| FMI age 14 | 3,192 | 0.33  (0.29, 0.36) | 2.72 x 10^-79^ | 2,233 | 0.30  (0.26, 0.34) | 3.97 x 10^-48^ | 3,192 | 0.61  (0.39, 0.92) | 3.39 x 10^-8^ | 3,192 | 0.07  (-0.16, 0.30) | 0.54 | 0.049 |
| FMI age 16 | 2,715 | 0.36  (0.32, 0.40) | 2.39 x 10^-83^ | 1,927 | 0.33  (0.29, 0.38) | 3.05 x 10^-50^ | 2,715 | 0.53  (0.32, 0.74) | 9.58 x 10^-7^ | 2,715 | -0.03  (-0.27, 0.22) | 0.84 | 0.001 |
| FMI age 18 | 2,430 | 0.34  (0.30, 0.38) | 1.55 x 10^-69^ | 1,739 | 0.32  (0.27, 0.37) | 4.24 x 10^-43^ | 2,430 | 0.54  (0.31, 0.77) | 3.01 x 10^-6^ | 2,430 | 0.02  (-0.23, 0.26) | 0.90 | 0.012 |

All results are the difference in mean offspring BMI in standard deviation (SD) units per greater SD of maternal pregnancy BMI. Model 1 multivariable regression with control for maternal age and offspring age and sex through standardisation of maternal BMI and offspring BMI; Model 2 multivariable regression additionally adjusted for parental social class, maternal and paternal education, parity and paternal BMI; Model 3: genetic instrumental variable (Mendelian randomization) with control for maternal age and offspring age and sex through standardisation of BMI; Model 4 genetic instrumental variable (Mendelian randomization) additionally adjusted for offspring allele score.
