# Supplementary material for: Using Genetic Variation to Explore the Causal Effect of Maternal Pregnancy Adiposity on Future Offspring Adiposity: A Mendelian Randomisation Study
Source: PLoS Med. 2017 Jan 24;14(1):e1002221. doi: 10.1371/journal.pmed.1002221 (PMC5261553; doi:10.1371/journal.pmed.1002221)
Supplement: S10 Table — (DOCX) [file pmed.1002221.s019.docx]

#### Supplementary Table 10 –Associations between maternal BMI and maternal BMI allele score and possible confounding factors in Generation R

| Variable |  | Maternal Body mass index (kg/m^2^) | | Maternal BMI allele score | | Maternal BMI allele score^†^ | |
| --- | --- | --- | --- | --- | --- | --- | --- |
|  | N | Coefficient*  (95% CI) | P | Coefficient* (95%CI) | P | Coefficient* (95%CI) | P |
| Household income (<1,600, 1,600-2,200, ≥2,200 **€)** | 2,001 | -0.10  (-0.15, -0.05) | 9.26 x 10^-5^ | 0.05  (0.00, 0.11) | 0.04 | -0.05  (-0.11, 0.02) | 0.14 |
| Maternal education (primary school, secondary school higher education) | 2,283 | -0.16  (-0.19, -0.13) | 6.34 x 10^-22^ | 0.04  (0.01, 0.07) | 0.02 | -0.03  (-0.06, 0.01) | 0.18 |
| Paternal education (primary school, secondary school higher education) | 1,654 | -0.12  (-0.15, -0.08) | 8.91 x 10^-11^ | 0.00  (-0.04, 0.04) | 0.98 | -0.04  (-0.08, 0.00) | 0.06 |
| Maternal smoking in pregnancy (never, early, throughout pregnancy) | 2,231 | 0.01  (-0.04, 0.07) | 0.67 | 0.04  (-0.01, 0.10) | 0.13 | 0.04  (-0.01, 0.10) | 0.14 |
| Maternal parity  (0, 1, 2, 3+) | 2,334 | 0.19  (0.14, 0.24) | 5.91 x 10^-13^ | -0.01  (-0.07, 0.04) | 0.61 | 0.02  (-0.03, 0.07) | 0.41 |
| Paternal BMI (kg/m^2^) | 1,875 | 0.06  (0.05, 0.07) | 1.16 x 10^-20^ | 0.00  (-0.02, 0.01) | 0.63 | -0.00  (-0.02, 0.01) | 0.60 |
| Ethnicity  (non-European, European) | 2,328 | -0.16  (-0.24, -0.08) | 7.19 x 10^-5^ | 0.33  (0.25, 0.41) | 6.66 x 10^-16^ | 0.06  (-0.11, 0.23) | 0.49 |

*Effect estimates are age-standardised maternal BMI and standardised allele scores per unit increase or category of the confounder

^†^additionally adjusted for ethnicity using the top 20 principal components obtained from PCA on offspring genome-wide data
