# Supplementary material for: Using Genetic Variation to Explore the Causal Effect of Maternal Pregnancy Adiposity on Future Offspring Adiposity: A Mendelian Randomisation Study
Source: PLoS Med. 2017 Jan 24;14(1):e1002221. doi: 10.1371/journal.pmed.1002221 (PMC5261553; doi:10.1371/journal.pmed.1002221)
Supplement: S11 Table — (DOCX) [file pmed.1002221.s020.docx]

#### Supplementary Table 11 – Simulation to investigate the direction and magnitude of bias in observational multivariable and instrumental variable regression models

| SIMULATION DETAILS |  | |  |
| --- | --- | --- | --- |
| N=10000,  10000 replications | Multivariable regression coefficient of outcome on maternal pre-pregnancy BMI | IV regression coefficient of outcome on maternal pre-pregnancy BMI without adjustment for offspring BMI | IV regression coefficient of outcome on maternal pre-pregnancy BMI with adjustment for offspring BMI |
| True effect = 0.05 | 0.70 | 0.25 | 0.03 |
