# Supplementary material for: Using Genetic Variation to Explore the Causal Effect of Maternal Pregnancy Adiposity on Future Offspring Adiposity: A Mendelian Randomisation Study
Source: PLoS Med. 2017 Jan 24;14(1):e1002221. doi: 10.1371/journal.pmed.1002221 (PMC5261553; doi:10.1371/journal.pmed.1002221)
Supplement: S12 Table — (DOCX) [file pmed.1002221.s021.docx]

#### Supplementary Table 12 – Association between transmitted and non-transmitted maternal haplotype scores and offspring BMI at age 7 in ALSPAC (Total N=3,720)

| Haplotype score | N | Coefficient* | SE | P |
| --- | --- | --- | --- | --- |
| 32-SNP maternal transmitted allele | 3,661 | 0.279 | 0.045 | 8.40x10^-10^ |
| 32-SNP maternal non-transmitted allele | 3,661 | 0.043 | 0.042 | 0.300 |
| 97-SNP maternal non-transmitted allele | 3,642 | 1.046 | 0.138 | 5.26x10^-14^ |
| 97-SNP maternal non-transmitted allele | 3,642 | 0.030 | 0.135 | 0.822 |

*Per-SNP increase in allele score
