# Supplementary material for: Using Genetic Variation to Explore the Causal Effect of Maternal Pregnancy Adiposity on Future Offspring Adiposity: A Mendelian Randomisation Study
Source: PLoS Med. 2017 Jan 24;14(1):e1002221. doi: 10.1371/journal.pmed.1002221 (PMC5261553; doi:10.1371/journal.pmed.1002221)
Supplement: S13 Table — (DOCX) [file pmed.1002221.s022.docx]

#### Supplementary Table 13 – Regression estimates for the application of MR-Egger and inverse-variance weighted methods for Mendelian randomization in ALSPAC and Generation R

| **Method** | **Coefficient** | **Estimate^a^ (SE)** | **95% CI** | **p-value** | **Heterogeneity between SNPs** | | |
| --- | --- | --- | --- | --- | --- | --- | --- |
| 1. **ALSPAC (n=32 SNPs)** | | | | | Q-test p-value | I^2^ |  |
| IVW | Slope (*β*) | 0.03 (0.10) | -0.15, 0.23 | 0.74 | 0.56 | 0 |  |
| MR-Egger | Slope (*β*) | -0.15 (0.16) | -0.46, 0.15 | 0.33 |  |  |  |
| MR-Egger | Intercept (*α*) | 0.012 (0.008) | -0.004, 0.029 | 0.13 |  |  |  |
| 1. **ALSPAC (n=97 SNPs)** | | | | | Q-test p-value | I^2^ |  |
| IVW | Slope (*β*) | 0.10 (0.07) | -0.04, 0.24 | 0.17 | 0.74 | 0 |  |
| MR-Egger | Slope (*β*) | -0.02 (0.11) | -0.24, 0.19 | 0.85 |  |  |  |
| MR-Egger | Intercept (*α*) | 0.006 (0.004) | -0.002, 0.015 | 0.15 |  |  |  |
| 1. **Generation R (n=32 SNPs)** | | | | | Q-test p-value | I^2^ |  |
| IVW | Slope (*β*) | 0.21 (0.12) | -0.02, 0.45 | 0.07 | 0.49 | 0 |  |
| MR-Egger | Slope (*β*) | 0.38 (0.19) | 0.01, 0.75 | 0.05 |  |  |  |
| MR-Egger | Intercept (*α*) | -0.012 (0.010) | -0.033, 0.009 | 0.27 |  |  |  |
| **Meta-analysis (ALSPAC and Generation R 32 SNPs)** | | | | | Q-test p-value | I^2^ |  |
| IVW | Slope (*β*) | 0.10 (0.08) | -0.05, 0.25 | 0.18 | 0.25 | 25 |  |
| MR-Egger | Slope (*β*) | 0.07 (0.12) | -0.17, 0.31 | 0.57 | 0.03 | 78 |  |
| MR-Egger | Intercept (*α*) | 0.003 (0.006) | -0.010, 0.015 | 0.67 | 0.06 | 72 |  |
| **Meta-analysis (ALSPAC 97 SNPs and Generation R 32 SNPs)** | | | | | Q-test p-value | I^2^ |  |
| IVW | Slope (*β*) | 0.13 (0.06) | 0.01, 0.25 | 0.03 | 0.43 | 0 |  |
| MR-Egger | Slope (*β*) | 0.08 (0.10) | -0.11, 0.27 | 0.07 | 0.07 | 70 |  |
| MR-Egger | Intercept (*α*) | 0.005 (0.004) | -0.001, 0.012 | 0.13 | 0.68 | 0 |  |

^a^ Units for IVW and MR-Egger slope coefficients represent the increase in offspring BMI for each unit increase in maternal BMI (adjusted for offspring genotype); MR-Egger intercept coefficients represent the average pleiotropic effect of a maternal genetic variant on offspring BMI (adjusted for offspring genotype).
