# Supplementary material for: Using Genetic Variation to Explore the Causal Effect of Maternal Pregnancy Adiposity on Future Offspring Adiposity: A Mendelian Randomisation Study
Source: PLoS Med. 2017 Jan 24;14(1):e1002221. doi: 10.1371/journal.pmed.1002221 (PMC5261553; doi:10.1371/journal.pmed.1002221)
Supplement: S14 Table — (DOCX) [file pmed.1002221.s023.docx]

*Supplementary Table 14 - Confounder adjusted multivariable associations of maternal pregnancy body mass index with offspring body mass index and fat mass index from age 7 to 18 in ALSPAC (Discovery sample).*

| Offspring outcome | Confounder^a^ adjusted multivariable regression results | | Confounder^b^ adjusted multivariable regression results | | Confounder^c^ adjusted multivariable regression results | |
| --- | --- | --- | --- | --- | --- | --- |
|  | N | Difference in mean offspring outcome (SD) per 1SD increase maternal BMI (95%CI) | N | Difference in mean offspring outcome (SD) per 1SD increase maternal BMI (95%CI) | N | Difference in mean offspring outcome (SD) per 1SD increase maternal BMI (95%CI) |
| BMI age 7 | 2,565 | 0.25 (0.21, 0.29) | 2,565 | 0.24 (0.20, 0.28) | 2,565 | 0.24 (0.21, 0.28) |
| BMI age 10 | 2,507 | 0.31 (0.27, 0.35) | 2,507 | 0.30 (0.27, 0.34) | 2,507 | 0.30 (0.26, 0.34) |
| BMI age 12 | 2,411 | 0.32 (0.29, 0.36) | 2,411 | 0.32 (0.28, 0.36) | 2,411 | 0.31 (0.28, 0.35) |
| BMI age 14 | 3,227 | 0.32 (0.28, 0.36) | 3,227 | 0.32 (0.28, 0.36) | 3,227 | 0.32 (0.28, 0.35) |
| BMI age 16 | 2,806 | 0.34 (0.30, 0.39) | 2,806 | 0.34 (0.30, 0.38) | 2,806 | 0.34 (0.29, 0.38) |
| BMI age 18 | 2,521 | 0.33 (0.28, 0.37) | 2,521 | 0.32 (0.28, 0.37) | 2,521 | 0.32 (0.27, 0.37) |
| FMI age 10 | 2,413 | 0.30 (0.26, 0.33) | 2,413 | 0.29 (0.25, 0.33) | 2,413 | 0.29 (0.25, 0.33) |
| FMI age 12 | 2,375 | 0.31 (0.27, 0.35) | 2,375 | 0.31 (0.27, 0.34) | 2,375 | 0.30 (0.26, 0.34) |
| FMI age 14 | 2,233 | 0.30 (0.26, 0.34) | 2,233 | 0.30 (0.26, 0.34) | 2,233 | 0.30 (0.26, 0.34) |
| FMI age 16 | 1,927 | 0.33 (0.29, 0.38) | 1,927 | 0.33 (0.28, 0.37) | 1,927 | 0.33 (0.28, 0.37) |
| FMI age 18 | 1,739 | 0.32 (0.27, 0.37) | 1,739 | 0.32 (0.27, 0.36) | 1,739 | 0.32 (0.27, 0.36) |

BMI: Body mass index; FMI: Fat mass index

In all analyses offspring body mass index has been standardized on their sex and age and maternal BMI has been standardised on her age

^a^ The following were adjusted for in multivariable regression analyses: parental social class, parental education, maternal smoking during pregnancy, parity and paternal BMI.

^b^ The following were adjusted for in multivariable regression analyses: parental social class, parental education, maternal smoking during pregnancy, parity, paternal BMI and offspring 97 SNP allele score.

^c^ The following were adjusted for in multivariable regression analyses: parental social class, parental education, maternal smoking during pregnancy, parity, paternal BMI and maternal 97 SNP allele score.
